# S1P regulates intervertebral disc aging by mediating endoplasmic reticulum–mitochondrial calcium ion homeostasis

**DOI:** 10.1172/jci.insight.177789

**Published:** 2024-11-08

**Authors:** Bingjie Zheng, Xuyang Zhang, Xiangxi Kong, Jie Li, Bao Huang, Hui Li, Zhongyin Ji, Xiaoan Wei, Siyue Tao, Zhi Shan, Zemin Ling, Junhui Liu, Jian Chen, Fengdong Zhao

**Affiliations:** 1 Department of Orthopaedic Surgery, Sir Run Run Shaw Hospital, Zhejiang University School of Medicine, Hangzhou, Zhejiang, China.; 2 Key Laboratory of Musculoskeletal System Degeneration and Regeneration Translational Research of Zhejiang Province, Hangzhou, Zhejiang, China.; 3 The First Affiliated Hospital of USTC, Division of Life Sciences and Medicine, University of Science and Technology of China, Hefei, Anhui, China.; 4 Department of Orthopaedic Surgery, Ningbo Medical Center Li Huili Hospital, Ningbo, China.; 5 Shenzhen Key Laboratory of Bone Tissue Repair and Translational Research, Department of Orthopaedic Surgery, The Seventh Affiliated Hospital of Sun Yat-sen University, Shenzhen, China.; 6 Department of Wound Healing, The First Affiliated Hospital of Wenzhou Medical University, Wenzhou, China.

**Keywords:** Aging, Bone biology, Bone disease

## Abstract

As the aging process progresses, age-related intervertebral disc degeneration (IVDD) is becoming an emerging public health issue. Site-1 protease (S1P) has recently been found to be associated with abnormal spinal development in patients with mutations and has multiple biological functions. Here, we discovered a reduction of S1P in degenerated and aging intervertebral discs, primarily regulated by DNA methylation. Furthermore, through drug treatment and siRNA-mediated S1P knockdown, nucleus pulposus cells were more prone to exhibit degenerative and aging phenotypes. Conditional KO of S1P in mice resulted in spinal developmental abnormalities and premature aging. Mechanistically, S1P deficiency impeded COP II–mediated transport vesicle formation, which leads to protein retention in the endoplasmic reticulum (ER) and subsequently ER distension. ER distension increased the contact between the ER and mitochondria, disrupting ER-to-mitochondria calcium flow and resulting in mitochondrial dysfunction and energy metabolism disturbance. Finally, using 2-APB to inhibit calcium ion channels and the senolytic drug dasatinib and quercetin (D + Q) partially rescued the aging and degenerative phenotypes caused by S1P deficiency. In conclusion, our findings suggest that S1P is a critical factor in causing IVDD in the process of aging and highlight the potential of targeting S1P as a therapeutic approach for age-related IVDD.

## Abstract 2

S1P plays a critical role in IVDD during the aging process, highlighting the potential of targeting S1P as a therapeutic approach for age-related IVDD

## Graphical abstract

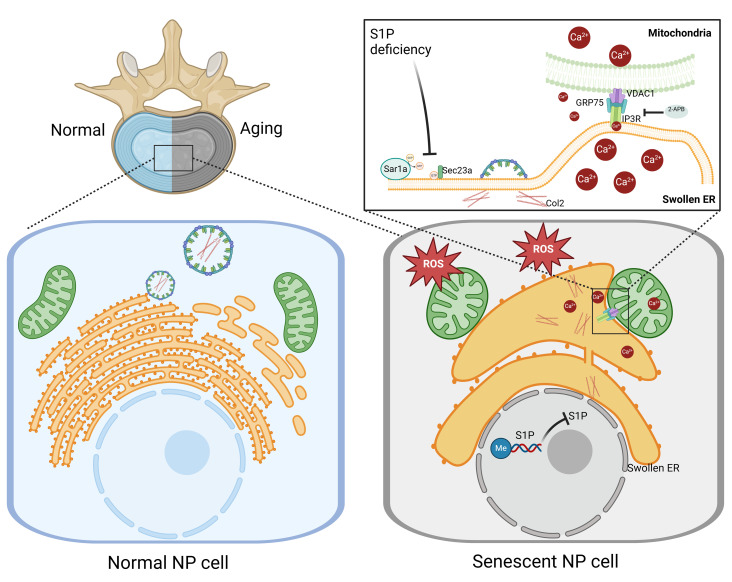

## Introduction

As the aging population continues to grow, aging-related diseases are becoming a pandemic (1). Among the health challenges, age-related intervertebral disc degeneration (IVDD) (2) stands out as one of the most significant chronic conditions. Aging intervertebral disc (IVD) is characterized by a significant reduction in the microvascular network of the cartilaginous endplate (CEP), leading to decreased thickness and altered permeability. Within this context, nucleus pulposus (NP) exhibit an aging-related secretory phenotype, with reduced anabolism and increased catabolism. These aging NP cells also secrete various inflammatory factors, further exacerbating the process of IVDD (3–5). Age-related IVDD can lead to the development of low back pain, affecting individuals’ quality of life and imposing a substantial economic burden on society (6). Therefore, gaining a comprehensive understanding of the biological mechanisms underlying IVD aging and developing therapeutic approaches holds crucial clinical significance in addressing age-related IVDD.

Site-1 protease (S1P) is a serine protease belonging to the proprotein convertase subtilisin/kexin family, also known as subtilisin/kexin-like protease-1 (SKI-1) (7, 8). Encoded by the membrane-bound transcription factor peptidase site 1 (MBTPS1) gene, S1P primarily localizes to the Golgi apparatus (9) and plays a crucial role in autophagy (10), lipid metabolism, and endoplasmic reticulum (ER) homeostasis (11, 12). Mice lacking S1P globally experience embryonic lethality prior to implantation (12). Moreover, S1P is essential for ECM signaling, axial elongation, and vertebral development during mitosis (13). Research has indicated that patients with mutations in the S1P gene exhibit skeletal developmental abnormalities and spinal deformities such as scoliosis (14). However, the effect of S1P on IVDs has not been investigated.

Cell senescence involves the gradual deterioration of tissue structure and function, encompassing cell apoptosis, decreased cell function, and inflammation within the tissue. Calcium homeostasis and mitochondrial oxidative damage are critical factors contributing to tissue degeneration (15). Aberrations in intracellular calcium ion homeostasis can lead to disruptions in cell signaling, energy metabolism, and antioxidant defense mechanisms, ultimately compromising cellular function and potentially triggering cell aging (16, 17). Oxidative damage can result in a decrease in mitochondrial membrane potential, impairments in the electron transport chain, and an increased generation of reactive oxygen species (ROS). These factors contribute to mitochondrial dysfunction, further accelerating the aging processes at the cellular and tissue levels (18, 19).

In this study, we investigated the role of S1P in the process of IVD aging and degeneration. Our findings reveal that S1P was downregulated in the process of IVD aging and degeneration due to increased DNA methylation level. The absence of S1P affected the process of cellular proteins transport, leading to abnormal ER swelling and subsequently resulting in increased ER-mitochondria contacts and calcium flux. This in turn led to mitochondrial oxidative stress and functional damage, ultimately contributing to IVD aging and degeneration. These findings suggest that S1P could be a target for improving and treating age-related IVDD.

## Results

### The expression of S1P decreases in degenerated and aged IVD.

**Figure 1:**
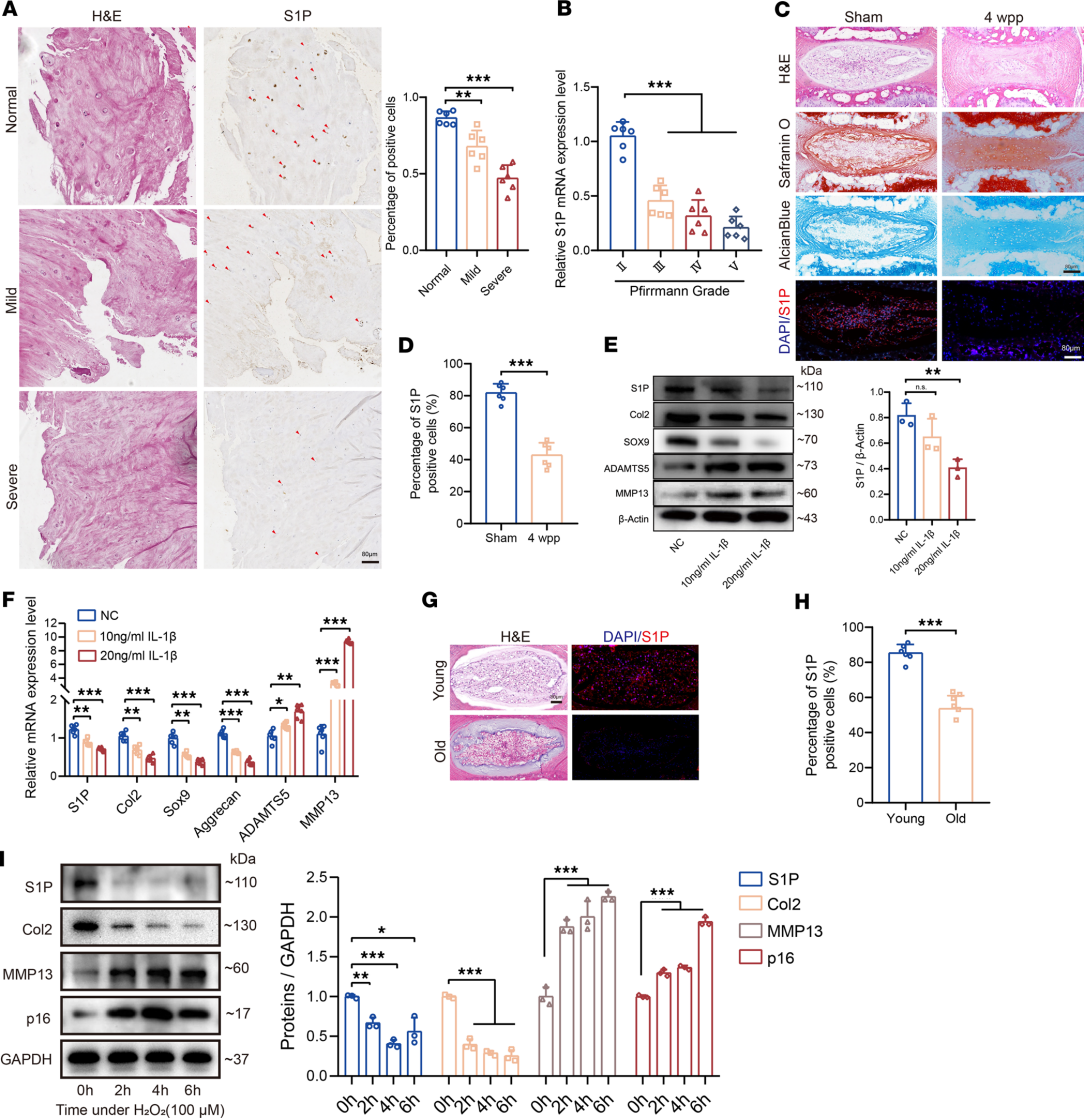
The expression of S1P in degenerated and aging IVD. (**A**) IHC was used to detect S1P expression in normal, mild, and severe degenerative IVD tissues and to determine the percentage of S1P^+^ cells (*n* = 6 per group; **P < 0.01, ***P < 0.001 compared with normal). Scale bar: 80 μm. (**B**) qPCR analysis of S1P gene expression in NP tissues from IVDs with varying Pfirrmann degeneration grades (*n* = 6; ***P < 0.001 compared with Pfirrmann II grade). (**C**) Representative images of Safranin O, Alcian blue, and S1P immunofluorescence staining in the sham and 4-week postpuncture groups (4 wpp). Scale bars: 80 μm. (**D**) Percentage of S1P^+^ cells in sham and 4 wpp groups (*n* = 6; ***P < 0.001 compared with sham). (**E**) Immunoblots of proteins in human NP cells exposed to IL-1β (10 ng/mL or 20 ng/mL for 48 h) and their quantification (*n* = 3; **P < 0.01 compared with NC). (**F**) Relative gene expression following IL-1β treatment (*n* = 6; *P < 0.05, **P < 0.01, ***P < 0.001 compared with NC). (**G**) Immunofluorescence images of S1P in young (6 weeks) and aged (24 months) mice. Scale bars: 80 μm. (**H**) Statistical analysis of S1P^+^ cells (*n* = 6; ***P < 0.001 compared with young). (**I**) Immunoblots of proteins in human NP cells treated with H_2_O_2_ for different time intervals, along with their quantitative analysis (*n* = 3; *P < 0.05, **P < 0.01, ***P < 0.001 compared with 0 h). Results are presented as means ± SD. Statistical significance was assessed using Student’s *t* test (**D**, **H**) or 1-way ANOVA (**A**, **B**, **E**, **F**, **I**), followed by Tukey’s post hoc analysis.

We collected clinical samples of human degenerated IVD tissues and observed that, within the normal subgroup, there was a higher expression of S1P. However, as the degree of degeneration increased (Figure 1A), the proportion of S1P immunopositive cells gradually decreased. We isolated NP cells from collected clinical samples of human degenerated IVDs and extracted RNA. The mRNA expression levels of S1P demonstrate a significant decrease in Pfirrmann Grades III, IV, and V degeneration groups when compared with the Pfirrmann Grade II degeneration group (Figure 1B).

To further validate the expression of S1P in degenerated IVDs, we established a mouse tail needle-punctured degeneration model. As shown in Figure 1C, in comparison with the sham group, mice subjected to the degeneration induction for 4 weeks exhibited a pronounced IVD degeneration phenotype; meanwhile, there was a significant, proportional reduction in the number of S1P immunopositive cells (Figure 1D). Subsequently, we induced degeneration in primary NP cells in vitro to assess the expression levels of S1P. The protein expression of S1P exhibited a significant decrease following IL-1β induction compared with the negative control (NC) group (Figure 1E). The quantitative PCR (qPCR) results confirm the trend observed in the protein blot (Figure 1F).

Additionally, we observed a decrease in the expression level of S1P in aging IVD tissues. Immunofluorescence analysis of young mice aged 6 weeks displayed widespread expression of S1P, whereas immunofluorescence analysis of naturally old mice at 24 months revealed a greater number of NP cells lacking immunopositivity for S1P (Figure 1, G and H). We observed that cell senescence progressively increased with passage number, and there was a concomitant decrease in S1P expression (Supplemental Figure 1A; supplemental material available online with this article; https://doi.org/10.1172/jci.insight.177789DS1). Moreover, we performed an in vitro validation of NP cell aging by inducing it with H_2_O_2_. Based on the results of the Cell Counting Kit-8 (CCK-8) assay (Supplemental Figure 1B), treatment with 200 μM H_2_O_2_ led to a significant decrease in cell proliferation activity, whereas treatment with 100 μM H_2_O_2_ preserved cell proliferation activity to a greater extent. Thus, we chose a concentration of 100 μm H_2_O_2_ to induce NP cell aging. We mapped the expression patterns of senescence-associated secretory phenotype (SASP) that exhibited differential expression after treating with 100 μm H_2_O_2_ for different hours followed by 5 days of continued culture to induce aging, and we observed that the expression of S1P significantly decreased both at the protein expression level (Figure 1I) and the mRNA level (Supplemental Figure 1, C–G). Together, S1P expression decreased in degenerated and aging IVD, both at the tissue and cellular levels, suggesting a potential link between S1P and disc degeneration.

### DNA methylation regulates the gene expression of S1P in aging NP cells.

**Figure 2:**
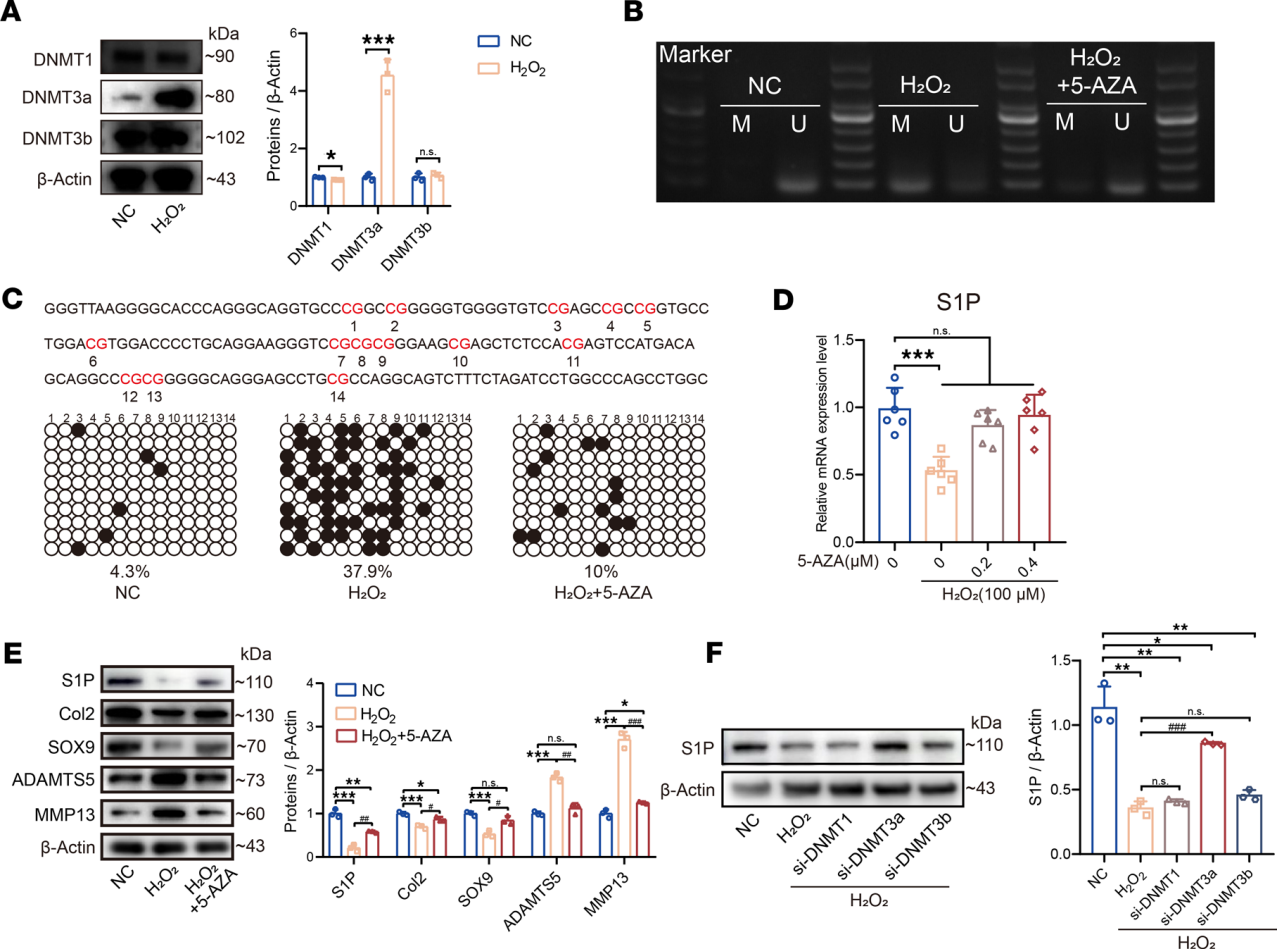
The expression of S1P is regulated by DNA methylation. (**A**) Immunoblots show methyltransferase expression in human NP cells after H_2_O_2_ treatment, along with quantification (*n* = 3 per group; *P < 0.05, ***P < 0.001 compared with NC). (**B**) MSP was performed on NP cells treated with H_2_O_2_ (100 μM) and rescued with 5-AZA (0.2 μM), indicating methylated (M) and unmethylated (U) forms of S1P. (**C**) BSP sequencing detected the S1P promoter region, with highlighted CpG islands in red. Unmethylated and methylated CpG sites are represented by white and black circles, respectively, for cells treated with H_2_O_2_ and rescued with 5-AZA. Different rows represent 10 bacterial monoclonals selected for sequencing in each group. (**D**) qPCR analysis of S1P expression in NP cells treated with H_2_O_2_ and rescued with varying 5-AZA concentrations (*n* = 6; ***P < 0.001 compared with negative treatment group). (**E**) Protein levels and quantification of S1P, Col2, SOX9, ADAMTS5, and MMP13 in NP cells treated with H_2_O_2_ and rescued with 5-AZA (*n* = 3; *P < 0.05, ***P < 0.001 compared with NC; ^#^*P* < 0.05, ^##^*P* < 0.01, ^###^*P* < 0.001 vs. H_2_O_2_ group). (**F**) Western blot analysis of S1P protein expression following H_2_O_2_ treatment, with DNMT1, DNMT3a, and DNMT3b knocked down by siRNA along with quantification (*n* = 3; *P < 0.05, **P < 0.01 compared with NC; ^###^*P* < 0.001 compared with H_2_O_2_). Results are expressed as means ± SD, analyzed by Student’s *t* test (**A**) or 1-way ANOVA (**D**–**F**), followed by Tukey’s post hoc test.

Based on literature, it has been found that the process of cellular aging is accompanied by diverse changes in DNA methylation. In certain tissues, specific genes exhibit high levels of methylation during aging, leading to a gradual reduction in their transcriptional levels and eventual gene silencing (20). Therefore, we hypothesize that gene expression of S1P was regulated through methylation mechanisms in aging NP cells. We observed that, after inducing aging in NP cells through H_2_O_2_ treatment, there were varying degrees of increased expression of the methylation transferases DNMT3a (Figure 2A). Subsequently, utilizing methylation-specific PCR (MSP) experiments, we found a significant increase in the gene methylation levels of S1P under H_2_O_2_ induction (Figure 2B). The brightness of the agarose gel band representing methylated S1P DNA (M) increased noticeably compared with the NC group, while the brightness of the agarose gel band representing unmethylated S1P DNA (U) weakened. Treatment with the methylation inhibitor 5-Azacytidine (5-AZA) led to a reduction in the brightness of the agarose gel band representing methylated S1P DNA (M). Furthermore, we performed Bisulfite Sequencing PCR (BSP) following sodium bisulfite treatment experiments to validate the methylation sites within S1P promoter region in aging NP cells. As shown in Figure 2C, based on computational predictions, there are 14 potential CpG islands within the S1P promoter region. In the NC group, only 4.3% of CpG islands in the S1P promoter were methylated, while after H_2_O_2_ treatment, 37.9% of CpG islands exhibited methylation. This provides evidence that the promoter region of S1P in aging NP cells experiences high levels of methylation, leading to reduced expression of the S1P. Treatment with 5-AZA resulted in a reduction in the methylation percentage of CpG islands.

After confirming the high methylation of the S1P promoter region in aging NP cells, we treated H_2_O_2_-induced aging NP cells with different concentrations of the methylation inhibitor 5-AZA. We found that it significantly rescued the downregulation of S1P in aging NP cells (Figure 2D). Additionally, we observed that, after rescuing the downregulation of S1P through 5-AZA treatment, there was a partial rescue of the degenerative phenotype in NP cells (Figure 2E).

To further elucidate which methylation transferases are responsible for the downregulation of S1P expression in aging NP cells, we employed siRNA to knock down different methylation transferases. We found that knocking down the methylation transferase DNMT3a significantly rescued the decrease in S1P levels after H_2_O_2_-induced aging (Figure 2F). Thus, in aging NP cells, the primary mechanism underlying the reduction of S1P is mediated by DNMT3a, which promotes DNA methylation of the S1P gene.

### The absence of S1P leads to IVD degeneration and premature aging.

**Figure 3:**
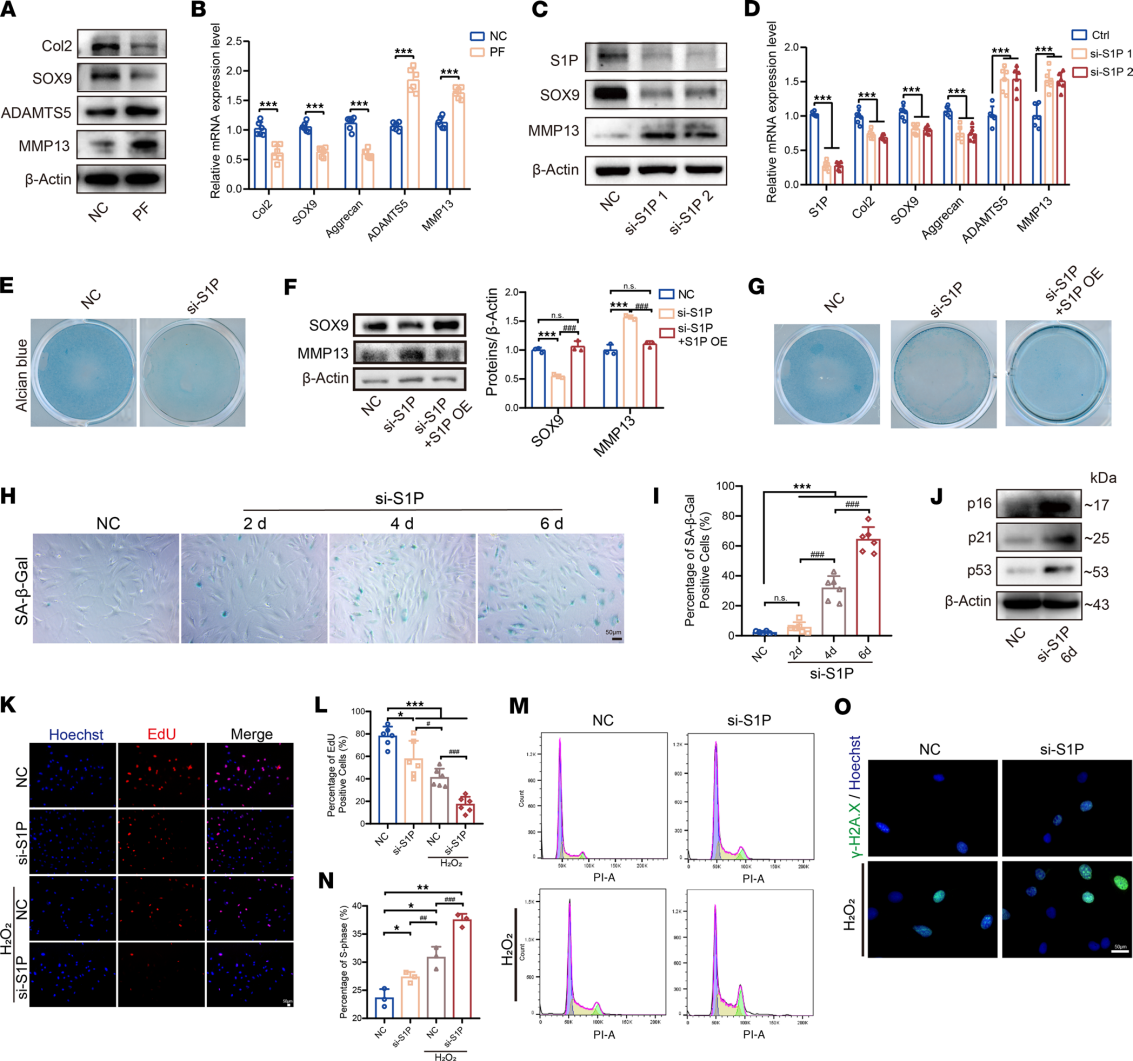
S1P deficiency leads to IVD degeneration and aging. (**A**) Immunoblots show gene expression in NP cells treated with PF. (**B**) qPCR analysis of these genes after PF treatment (*n* = 6 per group, ***P < 0.001 vs. NC group). (**C**) Immunoblots depict gene expression in NP cells following siRNA-mediated S1P knockdown. (**D**) qPCR results for gene expression after S1P knockdown (*n* = 6 per group, ***P < 0.001 vs. NC). (**E**) Alcian blue staining images of NP cells post-S1P knockdown. (**F**) Immunoblots illustrating gene expression after S1P knockdown and rescue by S1P overexpression (OE), with quantification (*n* = 3 per group, ***P < 0.001 vs. NC, ^###^*P* < 0.001 vs. si-S1P group). (**G**) Representative Alcian blue staining images after S1P knockdown and rescue by S1P OE. (**H**) SA–β-Gal staining images of NP cells after S1P knockdown and prolonged cultivation. (**I**) Percentage of SA–β-Gal^+^ cells (*n* = 6 per group, ***P < 0.001 vs. NC; ^###^*P* < 0.001 vs. 4d group). (**J**) Immunoblots for p16, p21, and p53 expression after S1P knockdown and 6 days of cultivation. (**K**) EdU staining and Hoechst labeling of nuclei across 4 groups. (**L**) Percentage of EdU^+^ cells (*n* = 6 per group, *P < 0.05, ***P < 0.001 vs. NC, ^#^*P* < 0.05, ^###^*P* < 0.001 vs. NC + H_2_O_2_ group). (**M**) Cell cycle analysis via flow cytometry across four groups. (**N**) Percentage of S phase (*n* = 3 per group, *P < 0.05, **P < 0.01 vs. NC, ^##^*P* < 0.01, ^###^*P* < 0.001 vs. NC + H_2_O_2_ group). (**O**) Representative immunofluorescence images of γ-H2A.X. Scale bars: 50 μm. Results are presented as mean ± SD, analyzed by Student’s *t* test (**B**) or 1-way ANOVA (**D**–**N**) with Tukey’s post hoc analysis.

Since the expression of S1P is downregulated in both degenerated and aging NP cells, further exploration is necessary to uncover the role of S1P in IVDs. We employed the specific inhibitor PF 429242 to suppress S1P function and found that it led to a decrease in anabolism markers (Col2 and SOX9) and an increase in catabolism markers (ADAMTS5 and MMP13) in NP cells (Figure 3, A and B). Meanwhile, we observed that the severity of the degenerative phenotype increased with higher concentrations of PF 429242 usage (Supplemental Figure 2, A–F). Although the PF 429242 inhibitor exhibits high selectivity for S1P, it could affect unknown targets. Hence, we further validated the effect of S1P on NP cells by specifically knocking down S1P using S1P siRNA. Upon S1P knockdown, similar to the trend observed with the PF 429242 inhibitor, NP cells exhibited a clear degenerative phenotype (Figure 3, C and D). Meanwhile, the knockdown of S1P resulted in decreased Alcian blue staining in NP cells (Figure 3E). Moreover, we performed a rescue experiment by transfecting an S1P overexpression plasmid. This rescue approach successfully restored the deficiency of S1P and was found to rescue the anabolic and catabolic processes in NP cells (Figure 3F). This rescue strategy improved the decreased Alcian blue staining observed in NP cells (Figure 3G).

Furthermore, During the process of cellular senescence, cells undergo DNA damage, decreased cell proliferation, and increased SA–β-Galactosidase (SA–β-Gal) activity (15). We examined these early markers of cellular senescence. We observed that, after S1P knockdown, the proportion of SA–β-Gal^+^ cells in NP cells increased over time (Figure 3, H and I). We analyzed the expression of aging-related markers in NP cells 6 days after siRNA knockdown of S1P and found that the expression levels of these markers increased compared with the NC group (Figure 3J).

We discovered that, after knocking down S1P, NP cells are more prone to exhibit an aging phenotype under the same conditions of H_2_O_2_ induction. Using EdU to detect cell proliferation levels, we observed a significant decrease in the proportion of EdU^+^ NP cells after both S1P knockdown and H_2_O_2_ treatment. However, among NP cells subjected to H_2_O_2_ treatment after S1P knockdown, the proportion of EdU^+^ cells exhibited a more pronounced decrease compared with either the S1P knockdown group or the H_2_O_2_ treatment group (Figure 3, K and L). This indicated that both individual S1P knockdown and individual H_2_O_2_ treatment affected the proliferation rate of NP cells, while S1P knockdown enhanced the effect of H_2_O_2_ on the proliferation activity of NP cells. Through flow cytometry analysis, we observed a significant increase in the proportion of cells in the S/G2 phase in the H_2_O_2_-induced group with S1P knockdown compared with the other groups, indicating cell cycle arrest (Figure 3, M and N). To further assess the cellular aging phenotype, we performed γ-H2A.X fluorescence staining to examine the DNA damage status. Compared with the NC group, NP cells with S1P knockdown exhibited a significant increase in γ-H2A.X fluorescence intensity under the same H_2_O_2_ treatment conditions (Figure 3O). In summary, the above experiments indicate that knocking down S1P sensitizes NP cells to the aging-inducing effects of H_2_O_2_, leading to a more pronounced manifestation of cellular aging phenotypes at an earlier stage. This suggests that S1P played a crucial role in regulating the response of NP cells to aging-related stressors.

### S1P–conditional KO mice exhibit a more severe degenerative phenotype and experience premature aging.

**Figure 4:**
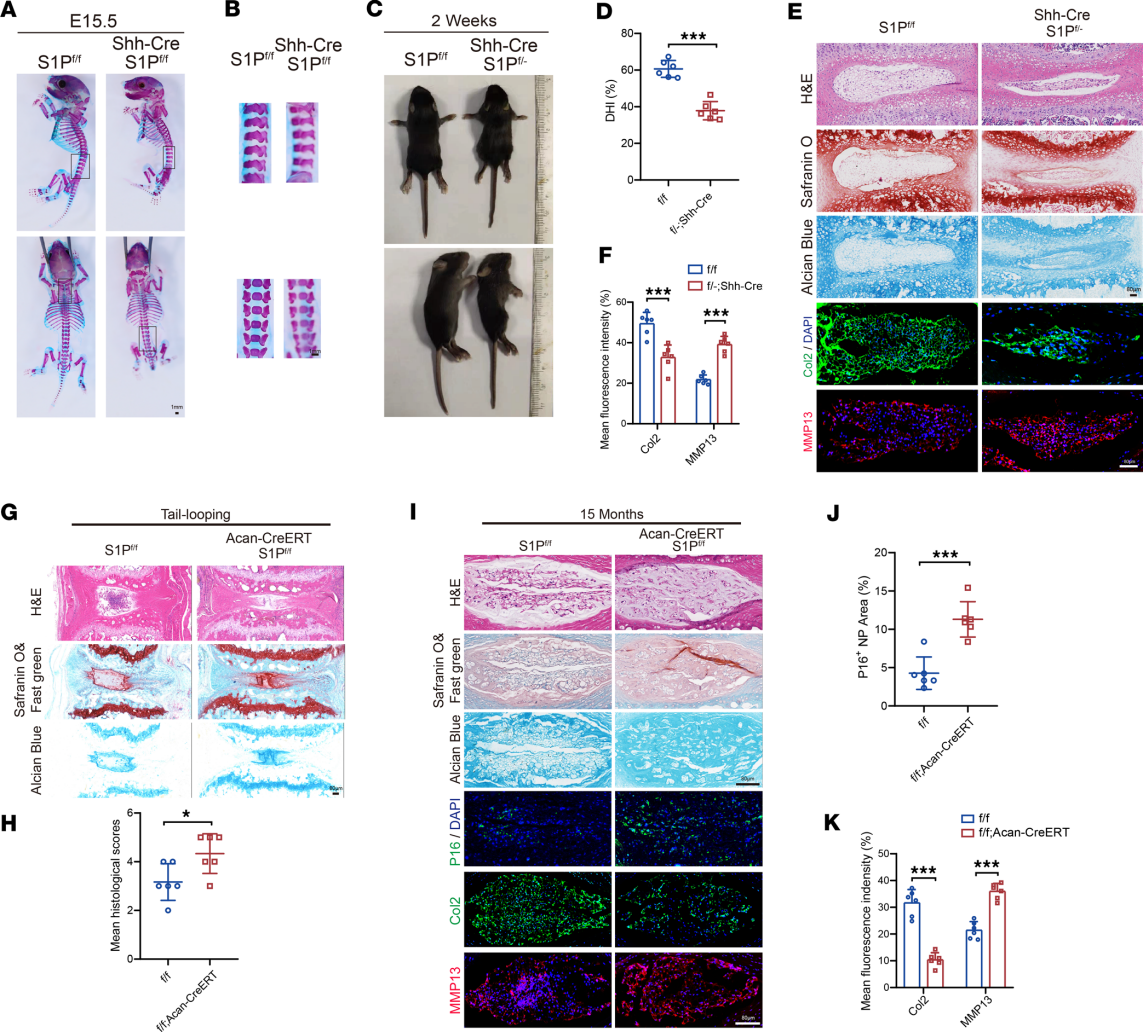
S1P-cKO mice exhibit a higher propensity for degeneration and aging. (**A** and **B**) Double-stained images of S1P^fl/fl^ and Shh-Cre-S1P^fl/fl^ embryonic mice at E15.5 days and enlarged images of the spinal region within the black frame (stained with Safranin O in red for bone and Alcian blue in blue for cartilage). Scale bar: 1 mm. (**C**) Images of 2-week-old S1P^fl/fl^ and Shh-Cre-S1P^f/-^ mice. (**D**) Statistical analysis of percentage of the IVD height index (DHI) for S1P^fl/fl^ and Shh-Cre-S1P^f/-^ mice (*n* = 6, each group) (**E**) Images of H&E, Safranin O, and Alcian blue staining of IVD tissues from 2-week-old S1P^fl/fl^ and Shh-Cre-S1P^f/-^ mice, as well as immunofluorescence images showing Col2 (green) and MMP13 (red) with DAPI staining for cell nuclei (Blue). (**F**) Mean fluorescence intensity for Col2 and MMP13 of 2-week-old S1P^fl/fl^ and Shh-Cre-S1P^fl/–^ mice (*n* = 6, each group). (**G**) H&E, Safranin O and Fast Green, and Alcian blue staining images of IVD tissues from 12-week-old S1P^fl/fl^ and Acan-CreERT-S1P^fl/fl^ mice after tail-looping modeling for 8 weeks. (**H**) Statistical analysis of the mean histological scores (*n* = 6, each group). (**I**) Images of H&E, Safranin O and Fast Green, and Alcian blue staining of IVD from 15-month-old naturally aging S1P^fl/fl^ and Acan-CreERT-S1P^fl/fl^ mice, as well as immunofluorescence images showing p16, Col2, and MMP13. (**J**) Percentage of p16^+^ area (*n* = 6, each group). (**K**) Mean fluorescence intensity for Col2 and MMP13 (*n* = 6, each group). Scale bars: 80 μm (**E**, **G**, and **I**). Results are shown as points with means ± SD. *P < 0.05, ***P < 0.001 compared with S1P^fl/fl^ mice. Student’s t-test or one-way ANOVA, followed by Tukey’s post hoc analysis, was employed to assess statistical significance.

To investigate the effect of S1P on IVD during growth and development, we established Shh-Cre-S1P^fl/fl^ mice. Unexpectedly, we found that homozygous Shh-Cre-S1P^fl/fl^ mice exhibited embryonic lethality. Alizarin red/Alcian blue–stained embryos demonstrate that the body segments of these homozygous Shh-Cre conditional KO (cKO) mice displayed a significantly reduced development length (Figure 4A). Furthermore, Alcian blue staining of the IVD region exhibited a noticeably lighter coloration in these homozygous Shh-Cre–cKO mice, indicating abnormal cartilage development (Figure 4B). Despite our discovery that Shh-Cre-S1P^fl/fl^ homozygous KO mice exhibit embryonic lethality, we did not observe significant pathological abnormalities in vital organs such as the heart and lungs of the heterozygous mice (Supplemental Figure 3A). Additionally, we used S1P immunofluorescence images to demonstrate the KO efficiency in NP tissue (Supplemental Figure 3B). Therefore, we speculate that the cause of embryonic lethality might be restricted somite development, which could affect the organogenesis of fetal mice.

Subsequently, when we cultured heterozygous S1P KO mice with Shh-Cre, the mice exhibited noticeable dwarfism. At the age of 2 weeks, their body length was shorter compared with the littermate control mice (Figure 4C). The Disc Height Index (DHI%) revealed that IVDD was notably restricted (Figure 4D). Meanwhile, through histological staining, we found a reduction in the NP area of the IVDs in the heterozygous KO mice (Figure 4E). Furthermore, there was a notable decrease in the mean fluorescence intensity of Col2, indicative of decreased anabolism and a significant increase of the mean fluorescence intensity of MMP13, indicative of catabolism (Figure 4, E and F).

To further investigate the influence of S1P on IVD degeneration and aging, we conditionally knocked out S1P by generating Acan-CreERT-S1P^fl/fl^ mice through the injection of tamoxifen for 5 consecutive days in 8-week-old mice. Meanwhile, we used S1P immunofluorescence images to demonstrate the KO efficiency in NP tissue (Supplemental Figure 3C). Subsequently, an IVD degeneration model by tail looping was established at age of 12 weeks. Compared with the littermate control group, the degenerative phenotype was more pronounced in the Acan-CreERT-S1P^fl/fl^ mice, with a significant difference in histological scores (Figure 4, G and H). This indicates that specific KO of S1P in IVD could accelerate the process of IVDD. Furthermore, we observed a significant increase in p16-immunopositive regions in Acan-Cre–cKO mice after 15 months of normal feeding (Figure 4, I and J). Additionally, these Acan-Cre–cKO mice exhibited a more pronounced SASP, including a significant decrease in Col2-immunopositive regions and a significant increase in MMP13-immunopositive regions (Figure 4, I and K). In conclusion, the results above indicate that the KO of S1P significantly affected the growth and development of IVDs and led to a more severe IVDD phenotype and an earlier manifestation of aging-related characteristics.

### The absence of S1P disrupts intracellular protein transport homeostasis in NP cells and leads to an increase in ER-mitochondria contacts.

**Figure 5:**
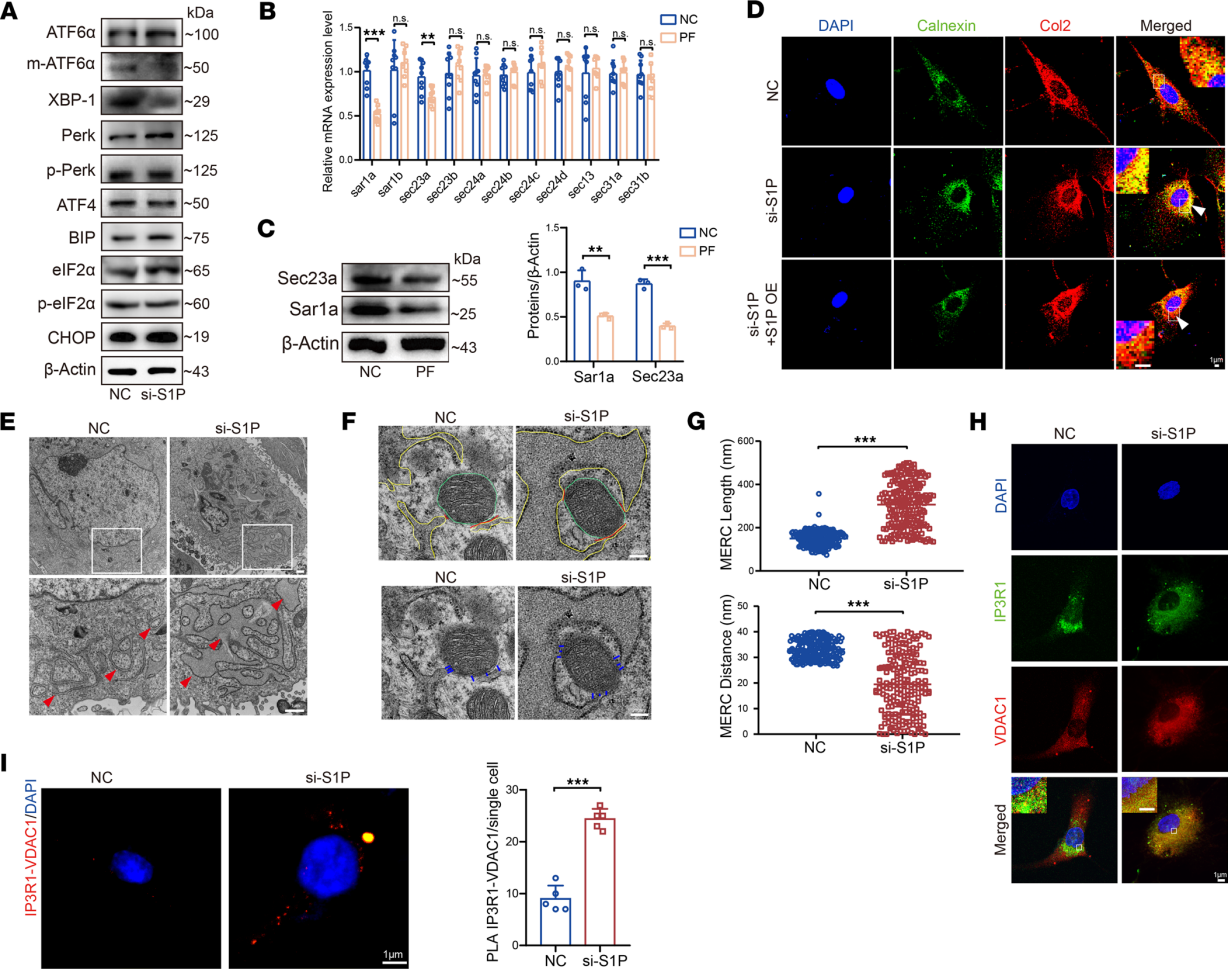
S1P deficiency affects ER state and ER-mitochondria–associated contacts. (**A**) Immunoblots depicting the genes expression related to ER stress in NP cells after S1P knockdown. (**B**) qPCR analysis for COP II–related gene expression. (**C**) Immunoblots depicting expression of Sec23a and Sar1a and quantification of the blot in NP cells treated with S1P inhibitor PF (*n* = 3, each group). The Sar1a blot provided is from the same sample as the Sec23a, and they were run contemporaneously. (**D**) Confocal immunofluorescence images of ER marker protein (Calnexin, green) and collagen protein (Col2, red) after S1P knockdown and rescue by S1P OE (white arrowheads indicate the yellow colocalization area). Scale bar: 1 μm. (**E**) Transmission electron microscopy (TEM) images depicting the ER state after S1P knockdown. Scale bar: 1 μm. Arrowheads indicate the ER. (**F** and **G**) TEM images showing MERC length and distance, with the yellow line representing the ER membrane, the green line indicating the mitochondrial membrane, the red line indicating length of MERC less than 50 nm between mitochondria and ER, and the blue line indicating distance of MERC less than 50 nm between mitochondria and ER as well as the statistical analysis (*n* = 200, each group). Scale bar: 200 nm. (**H**) Confocal immunofluorescence images of ER marker (IP3R1, red) and mitochondrial marker (VDAC1) after S1P knockdown. Scale bar: 1 μm. (**I**) Duolink proximity ligation assay (PLA) images and the statistical analysis of contact points per single cell after S1P knockdown. Red dots indicate IP3R1-VDAC1 contact points (*n* = 6, each group). Scale bar: 1 μm. Results are shown as means ± SD. **P < 0.01, ***P < 0.001 compared with NC group. Student’s *t* test, followed by Tukey’s post hoc analysis, was employed to assess statistical significance.

It has been known that S1P plays a significant role in ER stress and unfolded protein response. We mapped the expression patterns of representative genes from 3 pathways related to ER stress and unfolded protein response. We observed a reduction in the mature form of ATF6α (m-ATF6α) in the ATF6α pathway after knocking down S1P. The expression of XBP-1 in the IRE1α pathway also decreased. There was no significant change in ATF4, a component of the PERK pathway, or BIP, a molecular chaperone protein involved in the unfolded protein response (21). Furthermore, the expression of CHOP, a marker for late-stage ER stress–induced apoptosis, remained unchanged (Figure 5A). We confirmed this expression trend after treatment with different concentrations of the PF 429242 as well (Supplemental Figure 4A). CHOP has been confirmed as a signal for ER stress–induced apoptosis, inducing apoptosis under severe ER stress (22). This suggests that the absence of S1P primarily triggered early changes in ER stress, without reaching the level of severe stress that guides apoptosis. Therefore, the absence of S1P did not seem to induce IVD aging through ER stress. According to literature research, ATF6α and XBP-1 are involved in vesicular transport and COP II–related genes (23–25). Therefore, after inhibiting S1P with the inhibitor, we performed qPCR to assess the expression of COP II–related genes. We observed a downregulation in the gene expression of Sar1a and Sec23a (Figure 5B). The results from protein immunoblotting were consistent with the qPCR trends, showing a decrease in the expression of Sar1a and Sec23a upon S1P inhibition (Figure 5C). Also, we isolated human NP cells and conducted siRNA-mediated knockdown of S1P, followed by transcriptome analysis using next-generation sequencing (NGS) and identified negative regulation of COP II–related pathways through gene set enrichment analysis (Supplemental Figure 4B). These results suggest that the vesicular transport process in NP cells is affected upon S1P inhibition.

Subsequently, we conducted immunofluorescence double staining of ER marker protein (Calnexin) and collagen protein (Col2) to assess the transport of large molecular proteins in NP cells with S1P knockdown. As shown in Figure 5D, upon S1P knockdown, the increased yellow colocalization regions compared with the NC group indicated substantial retention of Col2 protein within the ER. Additionally, protein retention within the ER led to changes in cellular morphology, with cells appearing visibly swollen compared with the NC group. Conversely, upon S1P overexpression, the reduced yellow colocalization regions compared with the S1P knockdown group suggested a rescuing effect associated with S1P overexpression. Furthermore, we conducted electron microscopy to examine the ER status in NP cells after S1P knockdown and found that the ER in NP cells following S1P knockdown exhibited noticeable enlargement compared with the NC group (Figure 5E). This further supported the notion that the deficiency of S1P contributed to vesicular transport impairment, leading to protein retention within the ER and aberrant swelling of the ER.

At the same time, we observed that, upon knocking down S1P, the length of mitochondria-ER membrane contacts (MERC) significantly increased (Figure 5F). Additionally, the distance between mitochondria and ER membranes significantly decreased as did direct contact between mitochondria and ER (Figure 5G). Through immunofluorescence double staining using markers for ER (IP3R1) and mitochondria (VDAC1), we observed a significant increase in colocalization of immunofluorescence signals upon knocking down S1P (Figure 5H). Furthermore, we conducted a proximity ligation assay (PLA) to further validate the increased contact between the ER and mitochondria after knocking down S1P (Figure 5I).

**Figure 6:**
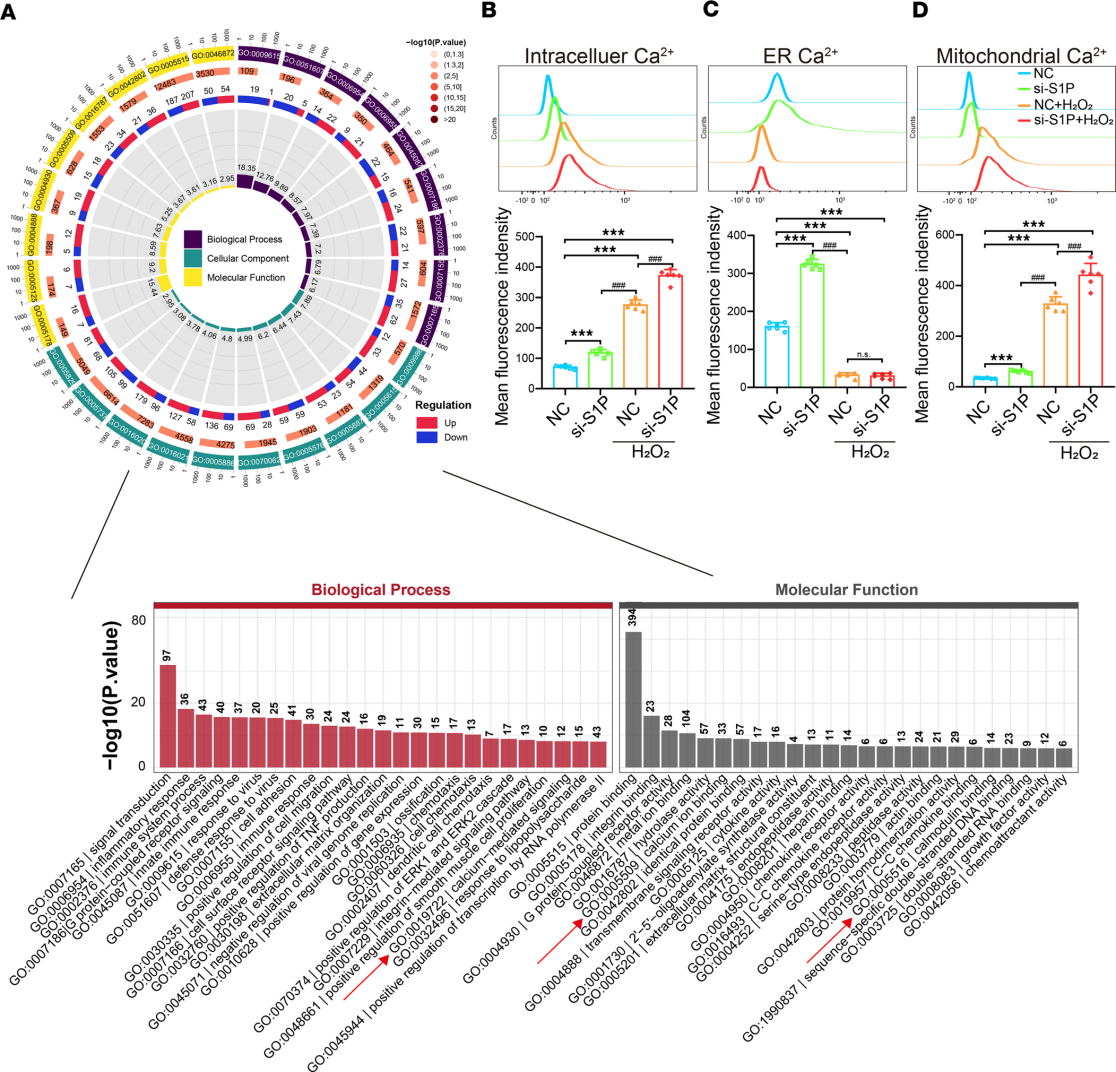
S1P deficiency affects calcium ion homeostasis in NP cells. (**A**) Next-generation gene sequencing (NGS) analysis of gene ontology (GO) pathways, with red arrows indicating the enriched calcium-related pathways. (**B**–**D**) Flow cytometry analysis of cytoplasmic calcium ions, ER calcium ions, and mitochondrial calcium ions, as well as statistical analysis of mean fluorescence intensity to represent calcium ion concentration of 4 groups. Results are shown as means ± SD. ***P < 0.001 compared with NC group. ^###^*P* < 0.001 compared with NC + H_2_O_2_ group. 1-way ANOVA, followed by Tukey’s post hoc analysis, was employed to assess statistical significance.

According to NGS data analysis, we observed enrichment of calcium-mediated signaling pathways and calcium-binding related pathways in NP cells after S1P knockdown (Supplemental Figure 4, C and D). The physiological activities and functions of the mitochondria and ER and are linked to calcium ion dynamics (Figure 6A). Consequently, we hypothesize that knocking down S1P affects intracellular calcium flux. As shown in Figure 6, B–D, NP cells in the physiological state exhibited relatively low levels of intracellular calcium ions, which moderately increased upon S1P knockdown. After induction with H_2_O_2_, there was a significant elevation in cytosolic calcium levels in NP cells, which was notably higher than both the NC group and the S1P siRNA group. Moreover, upon S1P knockdown and subsequent H_2_O_2_ induction, cytosolic calcium levels further rise, indicating a potentially heightened oxidative stress level (Figure 6B). The ER calcium levels in NP cells were relatively higher compared with cytoplasmic calcium levels, reflecting a physiological condition. However, upon S1P knockdown, ER calcium levels significantly increased. Previous studies have suggested that an increase in ER membrane content can expand the ER surface area, thereby enhancing its calcium storage capacity (26, 27). Thus, we hypothesized that the elevated ER calcium levels observed after S1P knockdown are due to ER expansion, resulting in enhanced calcium storage capacity. Upon H_2_O_2_ induction, the ER calcium levels in NP cells decreased, in accordance with prior research indicating that oxidative stress induced by H_2_O_2_ led to the opening of calcium channels on the ER membrane, promoting calcium efflux. Subsequently, following S1P knockdown and H_2_O_2_ induction, ER calcium levels within NP cells also significantly decreased (Figure 6C). Notably, the difference between the S1P knockdown and H_2_O_2_-induced groups is not significant, suggesting that the accumulated calcium in the ER following S1P knockdown was efficiently released in response to H_2_O_2_-induced oxidative stress. Despite a significant increase in mitochondrial calcium levels in S1P-knockdown NP cells compared with the NC group, the absolute calcium levels within mitochondria remained relatively low. Upon H_2_O_2_ induction, mitochondrial calcium levels in NP cells significantly increased due to oxidative stress–induced calcium efflux from the ER into the mitochondria. Furthermore, after S1P knockdown and subsequent H_2_O_2_ induction, mitochondrial calcium levels significantly rose compared with the H_2_O_2_-induced group (Figure 6D).

### Disrupted calcium flux leads to oxidative stress and mitochondrial dysfunction.

**Figure 7:**
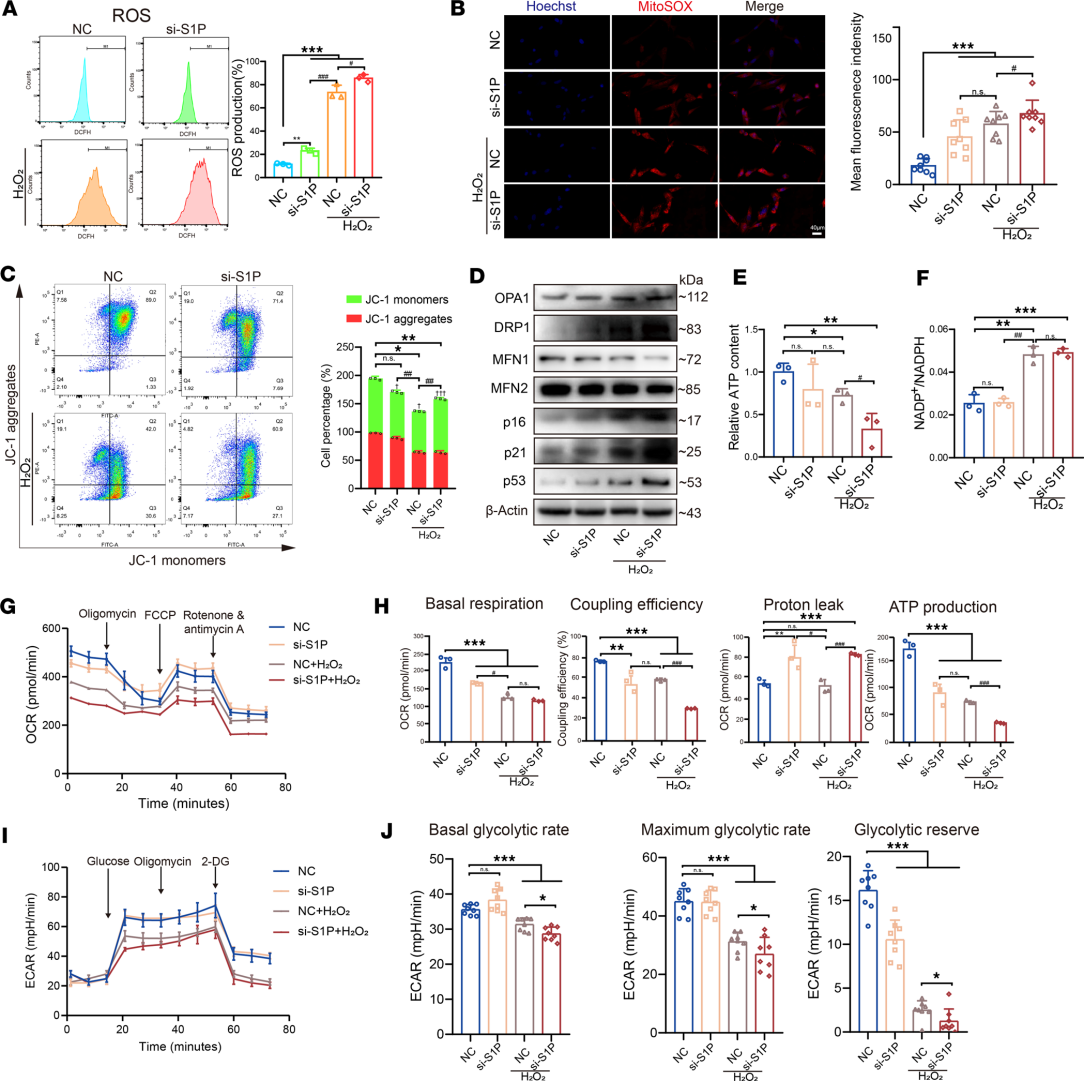
S1P deficiency affects mitochondrial function and energy metabolism. (**A**) Flow cytometry analysis of ROS levels and the statistical assessment of ROS production of 4 groups (*n* = 3, each group). (**B**) MitoSOX fluorescence images and the analysis of mean fluorescence intensity of 4 groups (*n* = 8, each group). Scale bar: 40 μm. (**C**) JC-1 staining for mitochondrial membrane potential (*n* = 3, each group), where green represents JC-1 monomers indicating low potential and red represents JC-1 aggregates indicating high potential. *P < 0.05, **P < 0.01 compared with NC group. ^##^*P* < 0.01 compared with NC + H_2_O_2_ group. †*P* < 0.05, †††*P* < 0.001 compared with JC-1 monomers and JC-1 aggregates. (**D**) Immunoblots depicting the expression of OPA1, DRP1, MFN1, and MFN2 as well as p16, p21, and p53. (**E**) Relative ATP content of 4 groups (*n* = 3, each group). (**F**) The ratio of NADP^+^/NADPH of 4 groups (*n* = 3, each group). (**G**) Measurement of oxygen consumption rate (OCR) by Seahorse experiment of 4 groups (*n* = 3, each group). (**H**) Statistical analysis of Seahorse experiment data, including basal respiration, maximal respiration, nonmitochondrial oxygen consumption, coupling efficiency, proton leak, and ATP production. (**I**) Measurement of extracellular acidification rate (ECAR) by Seahorse experiment of 4 groups (*n* = 8, each group). (**J**) Statistical analysis of Seahorse experiment data, including basal glycoltic rate, maximum glycolytic rate, and glycolytic reserve. Results are shown as means ± SD. *P < 0.05, **P < 0.01, ***P < 0.001 compared with NC group. ^#^*P* < 0.05, ^##^*P* < 0.01, ^###^*P* < 0.001 compared with NC + H_2_O_2_ group. One-way ANOVA, followed by Tukey’s post hoc analysis, was employed to assess statistical significance.

Elevated calcium signaling can activate enzymes producing ROS and generate free radicals (28). As depicted in Figure 7A, the percentages of ROS production in the S1P knockdown group, H_2_O_2_ treatment group, and S1P knockdown followed by H_2_O_2_ treatment group all exhibited significant increases. While the rise in ROS was relatively modest in the S1P knockdown group alone, with an approximate 11% increase compared with NC, it markedly escalated by 62% and 74% in the H_2_O_2_ treatment group and the S1P knockdown followed by H_2_O_2_ treatment group, respectively. These trends aligned with our previously measured mitochondrial calcium ion levels. We further validated mitochondrial oxidative stress in NP cells using MitoSOX staining. NP cells in the NC group exhibited relatively weak MitoSOX fluorescence intensity, while the strongest MitoSOX fluorescence intensity was observed in NP cells of the S1P knockdown, followed by H_2_O_2_ treatment group indicating a state of cellular stress (Figure 7B).

We then performed JC-1 experiments to assess mitochondrial membrane potential and observed that, in the NC group, the majority of NP cells exhibited JC-1 staining concentrated in the Q1 and Q2 quadrants, indicating higher mitochondrial membrane potential (Figure 7C). However, upon H_2_O_2_ induction, particularly after S1P knockdown followed by H_2_O_2_ treatment, a significant decrease in mitochondrial membrane potential was observed. Additionally, we noted that, in the NC group, S1P knockdown group, and H_2_O_2_-induced group, a subset of cells still clustered in the Q1 quadrant, suggesting the presence of NP cells with intact mitochondria. In contrast, in the S1P knockdown followed by H_2_O_2_ induction group, only a few cells remained in the Q1 quadrant, indicating a widespread reduction in mitochondrial membrane potential in NP cells. We examined proteins associated with mitochondrial morphology and found that the protein DRP1, which controls mitochondrial fission (29), was significantly increased in the group S1P knockdown followed by H_2_O_2_ treatment. Conversely, the protein MFN1, which controls mitochondrial outer membrane fusion (30), was significantly reduced. This suggests that there was less mitochondrial fusion and more fission in this group, ultimately leading to an increase in p16, p21, and p53 in NP cells (Figure 7D), which are well-known senescence marker genes. Additionally, electron microscopy revealed significant mitochondrial damage in the S1P knockdown followed by H_2_O_2_ induction group (Supplemental Figure 5A).

Subsequent energy metabolism analysis revealed that the group with S1P knockdown followed by H_2_O_2_ induction exhibited the lowest relative ATP content (Figure 7E). Furthermore, the NADP^+^/NADPH ratio results indicate a weakened antioxidant capacity in the S1P knockdown followed by H_2_O_2_ induction group (Figure 7F). To further elucidate the effect of S1P knockdown on NP cell energy metabolism, we employed the Seahorse XF-96 extracellular flux analyzer to measure the oxygen consumption rate (OCR) and extracellular acidification rate (ECAR) of cells. As illustrated in Figure 7, G and H, knocking down S1P significantly reduced the basal respiration, respiratory chain coupling efficiency, and ATP production. Knocking down S1P followed by H_2_O_2_ induction further decreased mitochondrial energy metabolism. It is noteworthy that the proton leak phenomenon was significantly elevated in the S1P knockdown group, indicating that, from the onset of S1P knockdown, mitochondria in the cells had already started to experience damage, with the potential to induce aging in NP cells. Meanwhile, knocking down S1P followed by H_2_O_2_ significantly reduced the basal glycolytic rate, maximum glycolytic capacity, and glycolytic reserve (Figure 7, I and J). Since NP cells are primarily glycolytic cells, this result indicates that knocking down S1P has an inhibitory effect on the energy metabolism of NP cells. Additionally, through NGS data analysis and GO enrichment, it was found that the pathways enriched in response to S1P knockdown were associated with aging (Supplemental Figure 5B). In summary, these data suggest that S1P knockdown exacerbated mitochondrial dysfunction and oxidative stress injury of NP cells, which reveals the mechanisms behind IVD aging caused by S1P deficiency.

### The calcium channel inhibitor 2-APB or the drug combination of dasatinib and quercetin can rescue IVD degeneration and aging.

**Figure 8:**
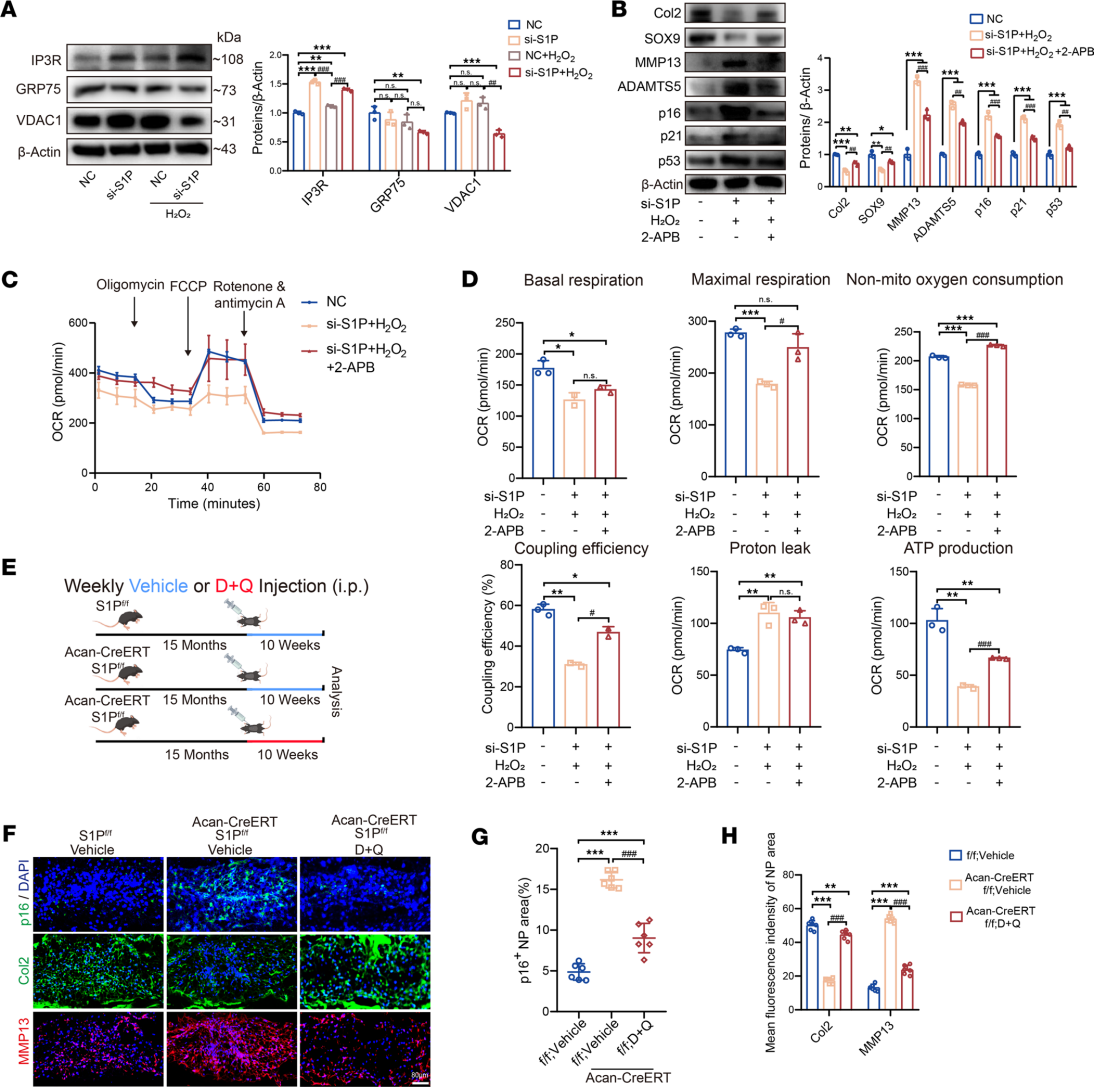
Calcium channel blockers and senolytic drugs can rescue the IVD aging caused by S1P deficiency. (**A**) Immunoblots showing calcium channel protein expression with quantification (*n* = 3). **P < 0.01, ***P < 0.001 vs. NC group; ^##^*P* < 0.01, ^###^*P* < 0.001 vs. NC + H_2_O_2_ group. (**B**) Immunoblots of senescence-related secretory phenotype genes in the si-S1P + H_2_O_2_ group and the 2-APB rescue group (*n* = 3). *P < 0.05, **P < 0.01, ***P < 0.001 vs. NC group; ^##^*P* < 0.01, ^###^*P* < 0.001 vs. si-S1P + H_2_O_2_ group. (**C**) Seahorse experiment OCR measurement for 3 groups (*n* = 3). (**D**) Statistical analysis of Seahorse data. *P < 0.05, **P < 0.01, ***P < 0.001 vs. NC group; ^#^*P* < 0.05, ^###^*P* < 0.001 vs. si-S1P + H_2_O_2_ group. (**E**) Schematic of peritoneal injections in 15-month-old mice for 10 weeks (vehicle or D + Q treatment). (**F**) Immunofluorescence of p16, Col2, MMP13 in S1P^fl/fl^ + vehicle, Acan-CreERT-S1P^fl/fl^ + vehicle, and D + Q groups. (**G**) Percentage of p16^+^ area (*n* = 6). (**H**) Mean fluorescence intensity of Col2 and MMP13 in 3 groups (*n* = 6). **P < 0.01, ***P < 0.001 vs. S1P^fl/fl^ + vehicle, ^###^*P* < 0.001 vs. Acan-CreERT S1P^fl/fl^ + vehicle group. Results as means ± SD, significance via 1-way ANOVA with Tukey’s post hoc analysis.

Considering the disruption of calcium flow from the ER to the mitochondria leading to mitochondrial dysfunction and eventually IVD aging, we conducted Western blot experiments and found that knocking down S1P led to an increase in the expression of the calcium channel protein IP3R, while other channel proteins, GRP75 and VDAC1, showed no significant changes (Figure 8A). In the S1P knockdown followed by H_2_O_2_ induction, there was a slight decrease in the calcium channel proteins GRP75 and VDAC1, which we speculate may be related to negative regulation after calcium flow disruption in NP cells. The upregulation of the calcium channel IP3R to some extent contributes to the calcium influx from the ER to the mitochondria. We then used the IP3R inhibitor, 2-aminoethoxydiphenyl borate (2-APB), to rescue NP cells subjected to S1P knockdown followed by H_2_O_2_ induction. Fortunately, the application of 2-APB effectively mitigated the degenerative phenotype observed in NP cells (Figure 8B). Additionally, the use of 2-APB to inhibit calcium ion channels also improved mitochondrial energy metabolism (Figure 8, C and D).

Furthermore, we employed dasatinib and quercetin (D + Q), a commonly used senolytic drug combination to reduce senescent cells (31). As illustrated in the schematic diagram (Figure 8E), we applied the D + Q treatment to naturally aging Acan-CreERT-S1P^fl/fl^ mice at 15 months of age for a total of 10 weeks, with a weekly i.p. injection. Our findings reveal that treatment with D + Q significantly reduced the presence of p16-immunopositive regions (Figure 8, F and G). Concurrently, treatment with D + Q aimed at eliminating senescent cells also exhibited a capacity to partially rescue the anabolism and catabolism of IVDs (Figure 8, F and H), suggesting its potential as a reliable therapeutic intervention for mitigating the aging effects resulting from S1P deficiency.

## Discussion

IVD aging is a complex process influenced by a multitude of factors (32, 33). While previous studies have identified genetic mutations in S1P that result in developmental abnormalities (13, 14), its specific role within IVDs remains unexplored. In this study, we have demonstrated that the absence of S1P leads to disruptions in intracellular protein trafficking, resulting in ER distention and subsequent abnormal ER-mitochondrial calcium flux. This ultimately triggers mitochondrial oxidative stress and functional impairment, culminating in the manifestation of IVD aging and degeneration phenotypes (Graphical Abstract).

S1P is known to activate various transcription factors, such as ATF6 and SREBPs. ATF6 is involved in regulating the transcription of genes related to ER stress, unfolded protein response, autophagy, and apoptosis (11). SREBPs participate in the regulation of genes associated with lipid metabolism, inflammation, and autophagy (34). ER stress has been shown to affect the metabolism of extracellular matrix–related (ECM-related) proteins, thereby contributing to IVDD (35). However, we did not observe widespread or severe ER stress following the knockdown of S1P, and only the activity of ATF6 directly activated by S1P, as well as XBP-1, exhibited significant decreases. Interestingly, ATF6A and XBP-1 have both been shown in previous studies to participate in the COP II vesicle trafficking process (23–25), and we validated the effect of S1P knockdown on Sar1a and Sec23a. Notably, prior studies have indicated that Sec23a can be transcriptionally regulated by ATF6α (24), which aligns with our findings. Sec23a functions in assembling cargo proteins from the ER into vesicles for transportation to the Golgi apparatus. This process involves the recognition and binding of specific signal sequences on cargo proteins, in coordination with the Sec24 and Sec13/31 subunits, leading to the formation of the COP II complex (36). This complex envelops cargo proteins for transportation from the ER to the Golgi apparatus. Sar1a, on the other hand, plays a role in cargo protein recognition and formation of the COP II complex during vesicular transport (37). Sar1a stimulates the exchange of GDP for GTP on the ER membrane, inducing conformational changes and subsequent recruitment of the Sec23/24 heterodimer (38). NP cells require the synthesis and secretion of collagen and other ECM molecules to maintain the structure and function of IVDs. When vesicular transport is restricted, in line with our research findings, large molecular proteins such as collagen could become trapped within the ER, leading to functional disruption in NP cells.

In mitochondria, the homeostasis of calcium ions plays a crucial role in both cellular physiology and pathology. It controls the rate of energy production in mitochondria and promotes the generation of ROS (28). In the presence of superoxide, highly reactive hydroxyl radicals are formed within cells, leading to damage of cellular proteins, RNA, DNA, and lipids, and under sustained stimuli, this can lead to cellular senescence phenotypes (39). Studies have indicated that the accelerated aging of IVD is associated with increased ROS production. Moreover, the antioxidant levels in degenerated NP cells are reduced, making them more susceptible to oxidative damage (40, 41). Through JC-1 experiments, we observed the most significant decrease in mitochondrial membrane potential in cells with S1P knockdown and H_2_O_2_ induction. Considering the calcium concentration gradient between the interior and exterior of mitochondria, the reduction in mitochondrial membrane potential can facilitate calcium influx. Moreover, the decrease in mitochondrial membrane potential can trigger the opening of the mitochondrial permeability transition pore (MPTP) (42), resulting in uncontrolled mitochondrial membrane permeability and calcium entry. Excessive calcium influx into mitochondria can also induce the opening of MPTP, leading to further reductions in mitochondrial membrane potential and increased ROS generation (43, 44). The increased calcium flow within mitochondria can be attributed to various factors. On one hand, as revealed in our research, we observed an increase in the expression of the calcium channel protein IP3R after S1P knockdown. On the other hand, previous studies have indicated that, when the distance between the ER and mitochondria is 12–24 nm or shorter, direct calcium transfer through concentration gradients can occur (27, 45, 46). Given that S1P knockdown leads to ER expansion and elevated calcium storage levels, the combined effect of increased ER calcium storage, ER-mitochondria calcium exchange, and direct calcium transfer contributes to a significant increase in calcium influx into mitochondria upon H_2_O_2_ induction, leading to a profound reduction in mitochondrial membrane potential. The reduction in mitochondrial membrane potential is an irreversible event in the early stages of apoptosis, which can lead to the release of cytochrome C, activation of the caspase protein family, impairment of mitochondrial respiratory chain, and decreased energy metabolism (47, 48). Additionally, mitochondrial damage can further elevate intracellular oxidative stress levels, causing damage to mitochondrial DNA; impair the synthesis of mitochondrial proteins; and inhibit mitochondrial respiration, thereby affecting normal mitochondrial function. Subsequently, this cascade of events can lead to mitochondrial apoptosis (49, 50), initiating a vicious cycle of events, ultimately, resulting in the appearance of senescence phenotypes in IVD (51). In summary, impaired mitochondrial calcium flux disrupts cellular calcium homeostasis, leading to increased ROS production from mitochondria. This oxidative stress, in turn, contributes to mitochondrial dysfunction, creating a feedback loop that exacerbates cellular damage and dysfunction IVD.

It is worth noting that knocking down S1P in the early stages (2 days) did not lead to a significant rise in the NP cell aging phenotype. We speculate that this might be because S1P’s regulation of NP cell aging is not directly mediated through protein-protein interactions or transcriptional regulation. Our experiments collectively indicate that, upon knocking down S1P, NP cells experienced ER swelling and accumulated more calcium ions. These calcium ions did not leak within the short term, thus not affecting cellular physiological functions. However, under other stress conditions, such as oxidative stress induced by H_2_O_2_, calcium ions within ER rapidly influxed into mitochondria, thereby exacerbating oxidative stress, inducing mitochondrial damage, and promoting NP cells eventual aging. Moreover, in vivo, with decreasing S1P expression during age process, various age-related stress factors emerged within the IVD, such as inflammatory stimuli and mechanical load. These factors further enhance S1P’s influence on IVD aging. We observed that S1P-KO mice tend to exhibit aging phenotypes more readily at the same age under normal feeding conditions, validating this hypothesis.

In conclusion, to our knowledge, our study provides the first elucidation of the role of S1P in the IVD and demonstrates that the depletion of S1P leads to IVD aging through vesicular transport, ER-mitochondrial calcium flux, and oxidative stress pathways. In the treatment of IVD aging, rescuing the decrease in S1P expression could mitigate the aging process caused by various stress factors within the IVD.

## Methods

Supplemental Methods are available online with this article.

### Sex as a biological variable

Sex was not considered as a biological variable in this research.

### Animals

S1P^fl/fl^ mice were supplied by Di Wang from Zhejiang University. Shh-Cre mice were a gift from An Qin from Department of Orthopaedics, Shanghai Key Laboratory of Orthopaedic Implant, Shanghai Ninth People’s Hospital, Shanghai Jiaotong University School of Medicine, Shanghai, China. Acan-CreERT mice were purchased from GemPharmatech. Acan-CreERT mice were induced by continuous i.p. injections of tamoxifen dissolved in corn oil (100 μg per gram of body weight) for 5 consecutive days at age of 8 weeks. Throughout all experiments, comparisons were made between the Shh-Cre-S1P^fl/fl^ or Acan-Cre-S1P^fl/fl^ mice and their S1P^fl/fl^ littermates.

### Animal model

The needle-puncture degeneration model was carried out in WT mice at age of 12 weeks. The mice were divided into 2 groups randomly: one was the sham group (no disc puncture) and the other group involved using a 27-gauge needle to puncture the Co7/Co8 and Co8–Co9 IVDs to the center. After 4 weeks, the mice were sacrificed, and samples were collected. The tail-looping degeneration model was performed in Acan-CreERT-S1P^fl/fl^ mice and S1P^fl/fl^ mice at the age of 12 weeks. Briefly, the tail of the mouse was fixed at a specific location, positioned between vertebrae Co5 and Co13 using a 0.8 mm stainless steel wire to secure the loop in place, and the end portion of the tail was removed (52). This disc-compression model was designed to ensure consistent compression in all mice, minimizing surgical variability. Mice were euthanized at 8 weeks following the looping procedure for histological assessment.

### IHC and immunofluorescence

Human IVD samples were sourced from Sir Run Run Shaw Hospital, Zhejiang University School of Medicine. Patient characteristics are listed in Supplemental Table 1. The IVD sections or cell slides underwent IHC or immunofluorescence staining. To block endogenous peroxidase activity, 3% H_2_O_2_ was applied for 10 minutes, succeeded by trypsin for 20 minutes and then 5% BSA for 30 minutes to prevent nonspecific antigen binding. Primary antibodies were left to incubate overnight at 4°C. On the subsequent day, sections were washed in PBS with 1% Tween and treated with corresponding HRP-conjugated secondary antibodies (Cell Signal Technology) for 1 hour at ambient temperature. Antibody information is in Supplemental Table 5. Diaminobenzidine (DAB) was used for immunolabeling visualization, followed by hematoxylin counterstaining.

For immunofluorescence staining, secondary antibodies labeled with Alexa Fluor 488 and/or 594 were applied for 1 hour at room temperature. See antibody information in Supplemental Table 5. The nucleus was stained using a 10 μg/mL solution of DAPI. Positively stained cells were quantified by assessing 3 randomly selected fields within the NP region, calculating the ratio of stained to total cells. Each specimen was sectioned at least 3 times, with data being averaged. Histological grading was used to evaluate cellular and morphological changes in both the annulus fibrosus (AF) and NP. Three independent and blinded investigators assessed the histological sections.

### Cell transfection

siRNA targeting S1P was purchased from Thermo Fisher Scientific, while siRNA targeting DNMT1, DNMT3a, and DNMT3b are provided in the Supplemental Table 3.

Human primary NP cells were plated in 6-well plates and cultured until reaching a cell confluence of 70%–80%. siRNAs were transfected into the NP cells using Lipofectamine 3000 (Invitrogen). Transfection reagent including 2 tubes. In 1 tube, 100 μL of Opti-MEM and 2 μg of siRNA per well were added. In the other tube, 100 μL of Opti-MEM and 5 μL of Lipofectamine 3000 per well were added. The 2 tubes stood for 2 minutes each and were then mixed them together and incubated at room temperature for 15 minutes. After replacing the medium in the culture plate with fresh medium, 200 μL of the transfection mixture to each well was added. Two days after transfections, the complete medium was exchanged for subculture.

### Induction of NP cell senescence in vitro

Before inducing with H_2_O_2_, a CCK-8 assay was conducted to determine the appropriate concentration (Supplemental Figure 1B). After confirming the healthy growth status of the passaged cells, cell culture plates were prepared according to the appropriate concentration determined through CCK-8 assays for NP cells. To induce NP cells senescence, the cells were treated with 100 μm H_2_O_2_ for induction, with durations of 2 hours, 4 hours, and 6 hours. Following the designated H_2_O_2_ induction times, cells were washed twice with PBS and fresh culture medium was added for an additional 5 days of cultivation.

As for treatment groups, human NP cells were randomly allocated into 4 groups: NC group (transfected with control siRNA), si-S1P group (transfected with S1P siRNA), H_2_O_2_ group (transfected with control siRNA for 2 days and treated with 100 μm H_2_O_2_ for 2 hours), and si-S1P + H_2_O_2_ group (transfected with S1P siRNA for 2 days and treated with 100 μm H_2_O_2_ for 2 hours).

### MSP experiment

The promoter sequence of the human S1P gene was acquired from the UCSC Genome Bioinformatics website (http://genome.ucsc.edu/) by selecting the upstream 2,000 bp region from the transcription start site. Subsequently, MSP primers were designed for the S1P promoter sequence using the methylation prediction website (http://www.urogene.org/methprimer/). The MSP primer sequences are listed in the Supplemental Table 4. One set of primers was designed for completely methylated specific sequences, capable of detecting unmethylated cytosines that remained unconverted during bisulfite treatment (human S1P MSP methylated primers: upstream [F] and downstream [R]). Another set of primers was designed for completely unmethylated sequences, targeting cytosines that were converted from unmethylated cytosines to uracils (human S1P MSP unmethylated primers F/R). The PCR products were subjected to 1% agarose gel electrophoresis, followed by visualization using a UV gel imaging system.

### BSP experiment

The promoter sequence of the S1P gene was submitted to the methylation prediction website to obtain BSP primer sets (Supplemental Table 4). DNA samples treated with sodium bisulfite were subjected to PCR amplification using the BSP primer sets. Subsequently, the purified PCR products were ligated into the pMD19-T plasmid vector and transformed into competent bacteria. The bacteria were plated on agar plates and incubated overnight. Then, 10 randomly selected colonies were amplified, and the samples were sent for gene sequencing. The sequencing results were aligned with the predicted CpG island sequence to identify methylated and unmethylated sites. The percentage of methylated sites in the cloned fragments was calculated.

### SA–β-Gal staining

After the experimental pretreatment of NP cells, the cell culture medium was removed and washed with PBS twice. In total, 1 mL of β-galactosidase staining fixative solution to each well was added and incubated at room temperature for 15 minutes. After fixation, the fixative solution was removed and the cells were washed with PBS for 3 minutes, repeating for 3 times. After discarding PBS, 1 mL of β-galactosidase staining working solution was added to each well, and cells were incubated at 37°C overnight. After overnight incubation, the staining solution was removed and washed with PBS for 3 minutes to remove excess staining solution; this was repeated 3 times. The cells were observed under a standard light microscope and the number of positively stained cells was counted.

### Cell cycle assay

The cell cycle distribution was assessed using the Cell Cycle Analysis Kit (Beyotime Biotech). To summarize, NP cells were collected, washed twice with cold PBS, and subsequently fixed in 70% ethanol at 4°C overnight. The cells were stained with a solution of PI and RNase A for 30 minutes at 37°C in the absence of light. Ultimately, The resulting cell cycle distributions were analyzed using flow cytometry (CytoFLEX) and interpreted with FlowJo V10 software to quantify the different cell cycle phases.

#### Quantitation of MERC.

MERC were determined based on regions with a distance less than 50 nm between the ER and the outer mitochondrial membrane (OMM) in transmission electron microscopy micrographs. Quantitative analysis involved assessing the length of MERC, and the mean distance less than 50 nm between ER and OMM within each MERC.

#### Duolink PLA.

Duolink PLA (Sigma-Aldrich) is a technique that allows for the detection, visualization, and quantification of protein interactions, typically at a scale of around 40 nm or less, as individual fluorescent dots under a microscope. Briefly, cells after treatment underwent permeabilization with 0.1% Triton X-100 and were subsequently incubated overnight at 4°C with primary antibodies (IP3R1 and VDAC1). See Supplemental Table 5 for full antibody list. Then the cells were washed with PBS containing 0.3% Tween and incubated with PLA probes. The ligation and polymerization steps were conducted in accordance with the manufacturer’s recommendations from Sigma-Aldrich. For analysis, at least 6 fields per group were acquired to count the dots and were normalize based on cell count with the BlobFinder software (Olink Bioscience).

#### Measurement of intracellular calcium ions.

Intracellular calcium ions were detected according the protocol (53). In brief, for cytoplasmic Ca^2+^ measurements, a 2.5 μM fluorescent Ca^2+^ indicator dye, Fluo-4AM (Yeasen Biotech), was used and incubated at 37°C for 30 minutes. For ER Ca^2+^ measurements, ionomycin (10 μM) was employed to clear intracellular free calcium ions, after which Fluo-4AM was used for measurement. For mitochondrial Ca^2+^ measurements, 2.5 μM Rhod-2 AM (Yeasen Biotech) was incubated at 37°C for 30 minutes. Finally, average fluorescence intensity was measured using flow cytometry (CytoFLEX).

### Measurement of intracellular ROS production

To assess ROS levels in NP cells, we employed the 2,7-dichlorofluorescin diacetate (DCFH-DA) assay (Yeasen Biotech). After treatment of each group, the cells were rinsed with PBS. Subsequently, a 10 μM solution of DCFH-DA was introduced to the plates and allowed to incubate for an additional 30 minutes at 37°C. Following this, the cells were washed with PBS and subjected to analysis using flow cytometry (CytoFLEX, Beckman Coulter).

### Mitochondrial membrane potential assay

Mitochondrial membrane potential assessment was performed using the JC-1 Assay Kit obtained from Yeasen Biotech, in accordance with the manufacturer’s instructions. In summary, NP cells were plated in 24-well plates and exposed to JC-1 staining solution in the absence of light at 37°C for 20 minutes. Following staining, the cells underwent 2 PBS washes and were subjected to analysis using flow cytometry (CytoFLEX). In total, 10,000 events were recorded during the flow cytometry process. At low mitochondrial membrane potential, JC-1 formed JC-1 monomer exhibiting green fluorescence, whereas the JC-1 aggregates show red fluorescence.

### Seahorse metabolic profiling assays

OCR measurements were conducted employing the Seahorse XF96 Mito Stress Test Kit and the Seahorse XF96 Extracellular Flux Bioanalyzer, both from Seahorse Bioscience. Following the treatment of each group, NP cells were seeded in XF96 Cell Culture miniplates, without phenol red. According to the protocol, this test involved the use of various compounds, including Oligomycin (an ATP-synthase inhibitor), FCCP (a mitochondria uncoupler; the FCCP concentration was 1 μM), Rotenone (a complex I inhibitor), and Antimycin A (a cytochrome C reductase inhibitor), all of which were obtained from MilliporeSigma. These compounds were used to create a profile of ATP-linked respiration, maximal respiration, and nonmitochondrial respiration. To standardize the results according to cell quantity in each well, the obtained OCR data were subjected to normalization through the Seahorse software. To determine the cell numbers, CyQuant (Invitrogen) was employed, as per the manufacturer’s guidelines. This quantification step was executed after the Seahorse measurements were concluded, directly on the Seahorse plate. The entire process was replicated 3 times at the biological level, and the measurements from these replicates were then merged for subsequent analysis.

### Statistics

The data were presented as mean ± SD. Prism 8 software (GraphPad Software) was utilized for data analysis. Two-tailed student’s *t* test or 1-way ANOVA, followed by Tukey’s post hoc analysis, was employed to assess statistical significance. At least 3 separate experiments were conducted, yielding consistent outcomes. Statistical significance was considered at *P* < 0.05.

### Study approval

The patient samples used in this study were collected with informed consent from the individuals and were approved by the Ethics Review committee of Sir Run Run Shaw Hospital. All animal model experiments adhered to the guidelines and protocols established in the *Guide for the Care and Use of Laboratory Animals* (National Academies Press, 2011), as well as the animal treatment standards of Zhejiang University (no. 25986).

### Data availability

The data analyzed to support the findings in this study are available from the Supporting Data Values file. Additional data related to this paper are available from a corresponding author upon request.

## Author contributions

BZ conceptualized experiments and contributed validation, investigations, data visualization, and writing. XZ contributed investigations (data collection, experiment design, and execution), methodology, and editing. XK contributed literature search and classification as well as editing. J Li contributed review and editing. BH contributed review and editing. HL contributed review and editing. ZJ contributed review and editing. XW contributed review and editing. ST contributed review and editing. ZS and ZL contributed review and editing, J Liu, JC, and FZ contributed conceptualization, project administration, funding acquisition, and review and editing.

## Supplementary Material

Supplemental data

Unedited blot and gel images

Supporting data values

## Version 1 09/24/2024

In-Press Preview

## Version 2 11/08/2024

Electronic publication
