# Supplementary material for: S1P regulates intervertebral disc aging by mediating endoplasmic reticulum–mitochondrial calcium ion homeostasis
Source: JCI Insight. 2024 Nov 8;9(21):e177789. doi: 10.1172/jci.insight.177789 (PMC11601718; doi:10.1172/jci.insight.177789)
Supplement: Supplemental data [file jciinsight-9-177789-s088.pdf]

Supplementary Methods

Cell culture and treatment experiment

Human primary NP cells were obtained from human surgical specimens. NP tissue can be distinguished from AF tissue based on tissue morphology. The gel-like NP tissues were digested with 0.2% type II collagenase (Sigma, St. Louis, MO, USA) overnight. The resulting cells were cultured in a complete culture medium (DMEM/F12, Gibco, Invitrogen, USA) supplemented with 10% fetal bovine serum (FBS, Gibco, Invitrogen, USA) and antibiotics, maintained in a 5% CO2, 37°C environment. The medium was refreshed every 2–3 days. Cells at passages 2 to 3 were used in the experiments.

Cell counting kit-8 assay (CCK8)

Cell suspensions of appropriate concentrations were added to each well of a 96-well plate and incubated at 37°C in a 5% CO2 cell culture incubator until they adhered and reached a confluence of 70%-80%. H_2O_2 concentrations were set at 50 μM, 100 μM, 200 μM, 400 μM, and 800 μM, along with a negative control (NC) group (without H_2O_2) and a blank control group (without CCK-8 solution). Each group was prepared with six replicate wells and added to the adherent cell culture plate. The 96-well plate was then incubated at 37°C in a 5% CO2 cell culture incubator for 24 hours.

H_2O_2 concentrations were set at 50 μM, 100 μM, 200 μM, 400 μM, and 800 μM, along with a negative control (NC) group (without H_2O_2) and a blank control group (without CCK-8 solution). Each group was prepared with six replicate wells and added to the adherent cell culture plate Cell suspensions of appropriate concentrations were added to each well of a 96-well plate and incubated at 37°C in a 5% CO2 cell culture incubator. H_2O_2 concentrations were set at 50 μM, 100 μM, 200 μM, 400 μM, and 800 μM, along

with a negative control (NC) group (without H_2O_2) and a blank control group (without CCK-8 solution). Each group was prepared with six replicate wells and added to the adherent cell culture plate. The 96-well plate was then incubated at 37°C in a 5% CO2 cell culture incubator for 24 hours.

After incubation, 10 μL of CCK-8 solution from the CCK-8 kit was added to each well (with care to avoid bubble formation in the wells to prevent interference with OD readings). The culture plate was then incubated in a cell culture incubator for 2 hours, with ongoing observation of color development. Subsequently, the optical density (OD) was measured at a wavelength of 450 nm using an enzyme-linked immunosorbent assay (ELISA) reader. The relative cell proliferation activity (%) was calculated using the formula: [OD (with H_2O_2) - OD (blank)] / [OD (without H_2O_2) - OD (blank)] × 100%. Finally, a graph was plotted with H_2O_2 concentration on the x-axis and relative cell proliferation activity percentage on the y-axis.

RNA extraction and RT-PCR analysis

NP cells obtained and cultured in 12-well plates with DMEM/F12 containing 10% FBS. Following degeneration induction using IL-1β or PF 429242, total RNAs were extracted using TRIzol reagent (Invitrogen, Carlsbad, CA, USA). Complementary DNA (cDNA) was synthesized from 1μg of RNA per sample using Takara kit (Shiga, Japan). RT-qPCR experiments were conducted using an ABI Prism 7500 system (Applied Biosystems, Foster City, CA, USA) with SYBR Green QPCR Master Mix (TakaraBio). The RT-qPCR reaction mixture included 5μL of SYBR Green QPCR Master Mix, 3μL of double-distilled water (ddH_2O), 1μL of cDNA, and 10 μM of both

forward and reverse primers. The primer sequences used are detailed in Supplementary Table 2. Cycle threshold (CT) data were collected and normalized to the housekeeping gene GAPDH. Relative CT values were calculated using the $2^{-\Delta\Delta CT}$ method.

Western blot analysis

NP cells obtained were lysed in RIPA lysis buffer containing 100 mM PMSF and phosphatase inhibitor, followed by centrifugation at 12,000 rpm for 15 minutes. Supernatants were collected and mixed with 1× loading buffer. Protein samples were resolved using 10% SDS-PAGE gels, transferred onto polyvinylidene fluoride (PVDF) membranes via electroblotting. The membranes were blocked with 5% skim milk dissolved in TBST buffer for 1 hour. PVDF membranes were divided based on different protein molecular weights and incubated overnight with primary antibodies all purchased from Abcam (Cambridge, MA, USA) at a dilution of 1:1000. Subsequently, the membranes were exposed to secondary HRP-conjugated IgG (Cell Signal Technology, 1:5000) for 1 hour at room temperature. Protein bands were visualized using enhanced chemiluminescence reagents (Amersham Biosciences, Buckinghamshire, United States) and the LAS-4000 Science Imaging System (Fujifilm, Tokyo, Japan). Image J software was employed for photo analysis.

Cell proliferation assay

Cell proliferation was assessed using EdU staining. NP cells were cultured in 24-well plates, each well containing 1 mL of culture medium and a sterile glass slide. The plates were pre-incubated in a 5% CO2 incubator at 37°C for 24 hours. Following transfection, a pre-warmed 20 μM EdU working solution was added to each well of the 24-well plate

and the cells were incubated for an additional 2 hours. After removing the culture medium, the cells were fixed with 1 mL of 4% paraformaldehyde at room temperature for 15 minutes. Subsequently, the cells were washed and treated with 1mL of 0.3% TritonX-100 in PBS per well for 10 minutes at room temperature, followed by a 0.5mL Click reaction for 30 minutes in the dark. After washing off the excess solution, the cells were incubated with 200μL of Hoechst 33258 solution at room temperature for 5 minutes in the dark and washed off the excess solution with PBS. Finally, fluorescence detection was performed.

Alcian Blue and Alizarin Red S Staining in fetal mice

The structural changes in cartilage during chondrocyte differentiation were assessed using Alcian blue staining, following established protocols. Additionally, Alizarin red staining was employed to examine the presence of ossified bone. In brief, the fetal masses were subjected to a 3-day fixation in formalin, followed by rinsing with PBS. Subsequently, they were stained with 1% acidic Alcian blue for 2 days, and then underwent five sequential washes with 70% ethanol to remove any residual dyes. This process resulted in cartilage regions appearing blue. Furthermore, the samples were counterstained by immersion in a 0.5% Alizarin red S solution for 2 days, causing the ossified bone regions to appear red.

Transmission electron microscopy (TEM) experiments

In preparation for transmission electron microscopy, cells underwent a fixation process: a 1:1 volume of 4% glutaraldehyde was added to the culture medium and incubated for 15 minutes at 4°C. Subsequently, the cells were fixed in 2% glutaraldehyde, washed

three times for 1 hour at 4°C, post-fixed with 2% OsO4 for 1 hour at 4°C, and dehydrated using an escalating ethanol gradient. Impregnation was achieved by using a mixture of Epon A (50%), Epon B (50%), and DMP30 (1.7%), followed by polymerization at 60°C for 72 hours. Ultrathin sections, approximately 70 nm thick, were cut using a UCT (Leica) ultramicrotome and mounted on 200 mesh copper grids. Staining was performed with uranyl acetate and lead citrate. Imaging was carried out using a Jeol 1400 JEM transmission electron microscope equipped with an Orius 600 camera and Digital Micrograph at the CIQLE platform (UCBL-Lyon).

Measurement of mitochondrial ROS production (MitoSOX)

Mitochondrial ROS generation levels were assessed using the MitoSOX assay according to protocol from Yeasen Biotech. In brief, NP cells were cultured in 24-well plates, containing a sterile glass slide. After treatment of each group, cells were incubated in a 5 μM MitoSOX reagent working solution at 37°C for 20 minutes. After two washes with PBS, the cells were then incubated with Hoechst 33258 solution at room temperature for 5 minutes in the dark, and washed off with PBS. Images were captured using an immunofluorescence microscope (BX51TRF; Olympus, Tokyo, Japan), and analysis was conducted using Image J software.

Measurement of ATP levels

The relative ATP content was quantified employing an ATP Assay Kit from Beyotime Biotech. Cellular lysis was achieved by employing ATP lysis buffer, followed by centrifugation at 12,000 g and 4°C for 5 minutes. The working solution was initially placed in 96-well plate, and subsequently, the samples were introduced into the

detection well. The relative light units were determined with a luminescence microplate reader (Awareness Technology, Inc., USA).

Measurement of $NADP^+$/NADPH ratio

The $NADP^+$/NADPH ratio was determined using an $NADP^+$/NADPH quantification kit following the protocol from Beyotime Biotech. Briefly, the $NADP^+$/NADPH assay relies on a glucose dehydrogenase cycling reaction in which NADPH, generated in the reaction, reduces a probe to yield a highly fluorescent product. The $NADP^+$/NADPH ratio was determined using the following formula: $NADP^+$/NADPH ratio = (NADP total − NADPH)/NADPH.

Supplementary Table 1

Summary of human intervertebral disc specimens (12 IVDs).

Degeneration	Pfirrmann grade	Age/Gender	Vertebral Level
Normal	Grade I	53/M	L4/L5
	Normal	21/F	L5/S1
	Grade I	51/M	L4/L5
	Grade I	54/M	L4/L5
Mild	Grade II	56/F	L3/4
	Grade II	56/F	L4/L5
	Grade III	62/F	L1/2
	Grade II	57/M	L4/L5
Sever	Grade IV	67/F	L5/S1
	Grade IV	56/F	L4/5
	Grade V	55/F	L1/2、L5/S1
	Grade IV	60/M	L5/S1

Supplementary Table 2

The forward (F) and reverse (R) nucleotide sequences of specific gene PCR primers.

PCR Primers	Sequence (5’ - 3’)
Human S1P F	TACGGGAAGGCATTCGAGC
Human S1P R	CAGCAACTCTTACATTAGCACCT
Human Col2 F	TGGACGCCATGAAGGTTTTCT
Human Col2 R	TGGGAGCCAGATTGTCATCTC
Human SOX9 F	AGCGAACGCACATCAAGAC
Human SOX9 R	CTGTAGGCGATCTGTTGGGG
Hm-Aggrecan F	ACTCTGGGTTTTCGTGACTCT
Hm-Aggrecan R	ACACTCAGCGAGTTGTCATGG
Hm-ADAMTS5 F	ACTACGATGCAGCTATCCTGT
Hm- ADAMTS5 R	GTCCCAACGTCTGCCATTC
Hm-Aggrecan F	TCCTGATGTGGGTGAATACAATG
Hm-Aggrecan R	GCCATCGTGAAGTCTGGTAAAAT

Supplementary Table 3

The sequences of siRNA.

Oligos	Sequence (5’ - 3’)
Human si-DNMT1	GGAATGGCAGATGCCAACAGC
Human si-DNMT3a	CTACTACATCAGCAAGCGCAA
Human si-DNMT3b	AGATGACGGATGCCTAGAGTT

Supplementary Table 4

The sequences of BSP and MSP.

Oligos	Sequence (5’ - 3’)
Human S1P BSP F	GGGTTAAGGGGTATTTAGGGTAGGTGT
Human S1P BSP R	ACCAAACTAAACCAAAATCTAAAAAAACTA
Human S1P MSP M F	TACGAGTTTATGATAGTAGGTTCGC
Human S1P MSP M R	AACAAAATAAAAACGAAAAAACGTA
Human S1P MSP U F	TGAGTTTATGATAGTAGGTTTGTGG
Human S1P MSP U R	AACAAAATAAAAACAAAAAAACATA

Supplementary Table 5

The information for antibodies.

Product Name	Dilution Factor	Source	Catalogue
MBTPS1	1000	Invitrogen	PA5-101662
Col2	1000	Santa Cruz	Sc52658
SOX9	1000	Cell signaling technology	82630T
ADAMTS5	1000	Abcam	Ab41037
MMP13	1000	Cell signaling technology	69926T
ACAN	1000	Cell signaling technology	28971T
p16	1000	Cell signaling technology	18769T
p21	1000	Abcam	Ab109199
p53	1000	Cell signaling technology	9282T
DNMT1	1000	Abcam	Ab188453
DNMT3a	1000	Cell signaling technology	32578T
DNMT3b	1000	Cell signaling technology	67259T
ATF6α	1000	Abcam	Ab122897
XBP-1	1000	Cell signaling technology	12782T
Perk	1000	Cell signaling technology	5683T
p-Perk	1000	Abcam	Ab192591
ATF4	1000	Cell signaling technology	97038T
BIP	1000	Cell signaling technology	3177T
eIF2α	1000	Abcam	Ab169528
p-eIF2α	1000	Cell signaling technology	3398T
CHOP	1000	Cell signaling technology	2895T
Sec23a	1000	Cell signaling technology	8162S
Sar1a	1000	Abcam	Ab125871
OPA1	1000	Cell signaling technology	67589T
DRP1	1000	Cell signaling technology	8570T
MFN1	1000	Cell signaling technology	14739T
MFN2	1000	Cell signaling technology	9482T
IP3R	200	Santa Cruz	Sc271197
GRP75	1000	Cell signaling technology	2816S
VDAC1	200	Cell signaling technology	4866T
GAPDH	1000	Cell signaling technology	2118T
β-Actin	1000	Cell signaling technology	4967S
Alexa Fluor 488	100	Cell signaling technology	4412S
Alexa Fluor 594	100	Cell signaling technology	4409S
Rabbit IgG	1000	Cell signaling technology	7074
Mouse IgG	1000	Cell signaling technology	7076

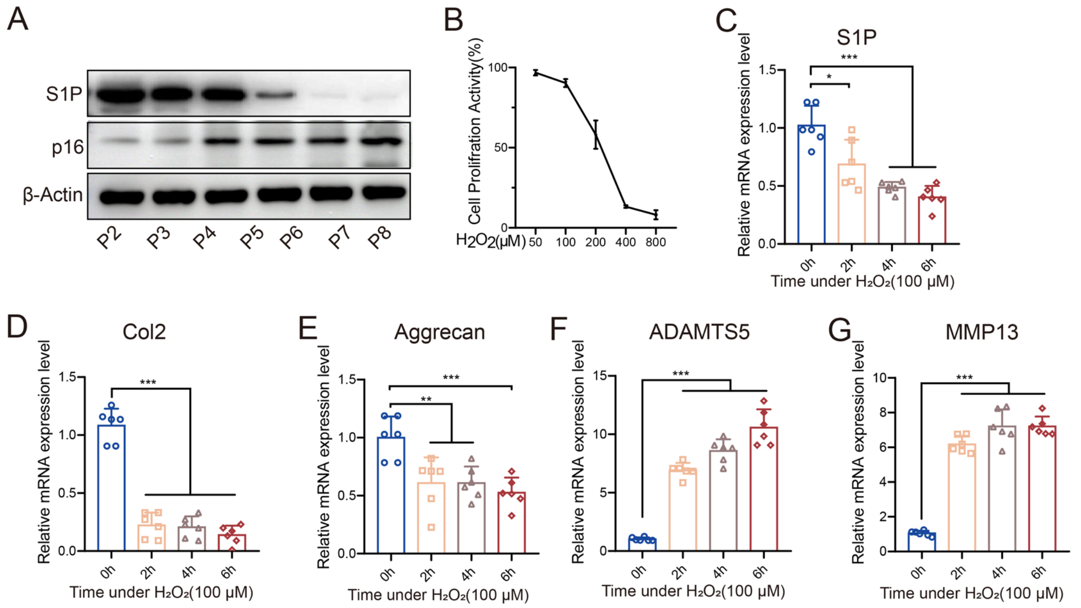

Supplemental Figure 1

(A) Immunoblots showing the gene expression of S1P and p16 in primary NP cells of different passages. **(B)** Percentage of cell proliferation activity of human NP cells under different concentrations of H_2O_2 treatment as measured by CCK-8 assay. **(C - G)** qPCR analysis of S1P, Col2, Aggrecan, ADAMTS5 and MMP13 of NP cells under different concentrations of H_2O_2 treatment (n = 6, each group). * $p < 0.05$, ** $p < 0.01$, *** $p < 0.001$ compared with 0h group. Results are shown as means ± SD. Student's t-test or one-way ANOVA, followed by Tukey's post hoc analysis, was employed to assess statistical significance.

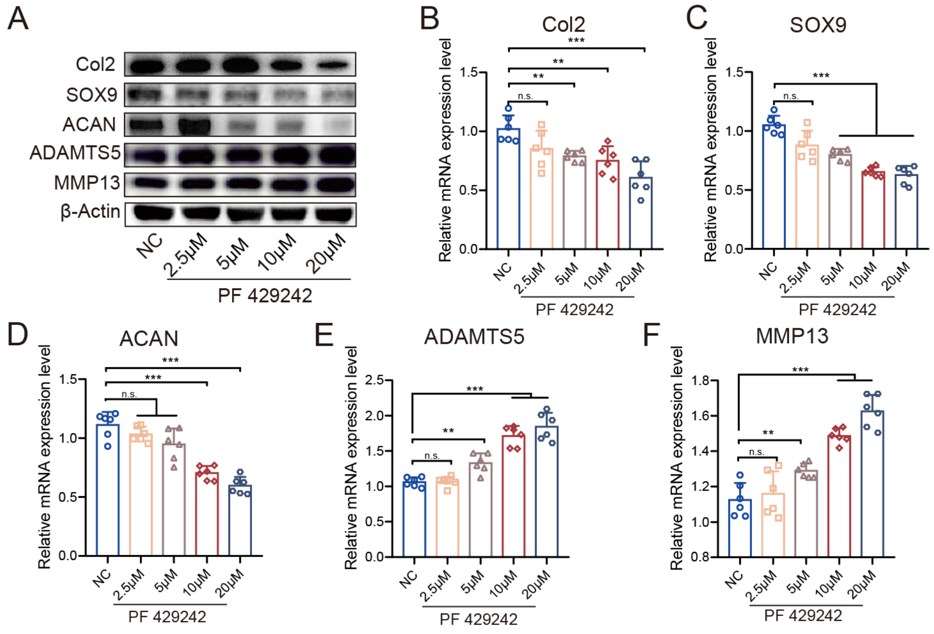

Supplemental Figure 2

(A) Immunoblots showing the gene expression of Col2, SOX9, Aggrecan, ADAMTS5 and MMP13 in NP cells treated with different concentration of PF 429242. **(B - F)** qPCR analysis of Col2, SOX9, Aggrecan (ACAN), ADAMTS5 and MMP13 in NP cells treated with different concentration of PF 429242 (n = 6, each group). ** $p < 0.01$, *** $p < 0.001$, n.s = no significant compared with NC group. Results are shown as means ± SD. Student's t-test or one-970 way ANOVA, followed by Tukey's post hoc analysis, was employed to assess statistical significance.

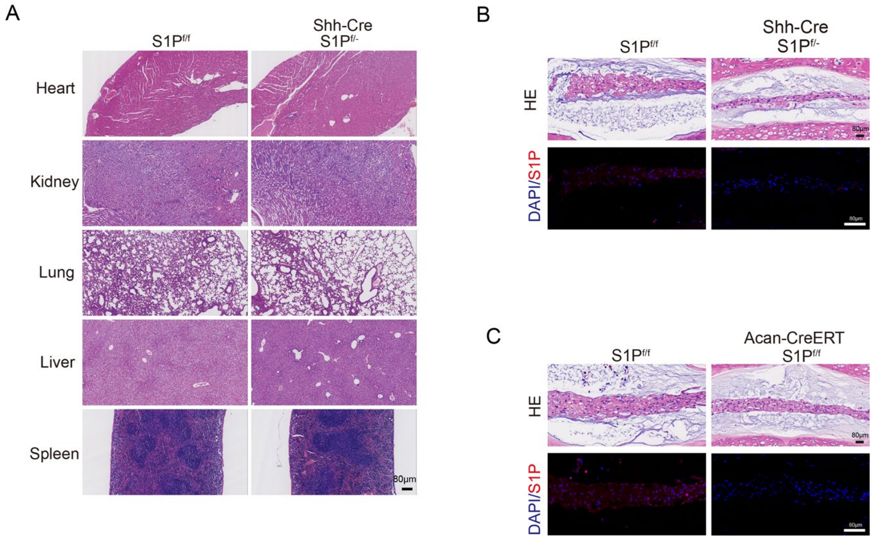

Supplemental Figure 3

(A) H&E images of the heart, kidney, lung, liver, and spleen from S1P$^{f/f}$ mice and Shh-Cre-S1P$^{f/f}$ mice. **(B, C)** Representative immunofluorescence images of S1P in S1P$^{f/f}$, Shh-cre-S1P$^{f/-}$ and Acan-creERT-S1P$^{f/f}$ mice at 8 weeks old. Scale bars: 80 μm.

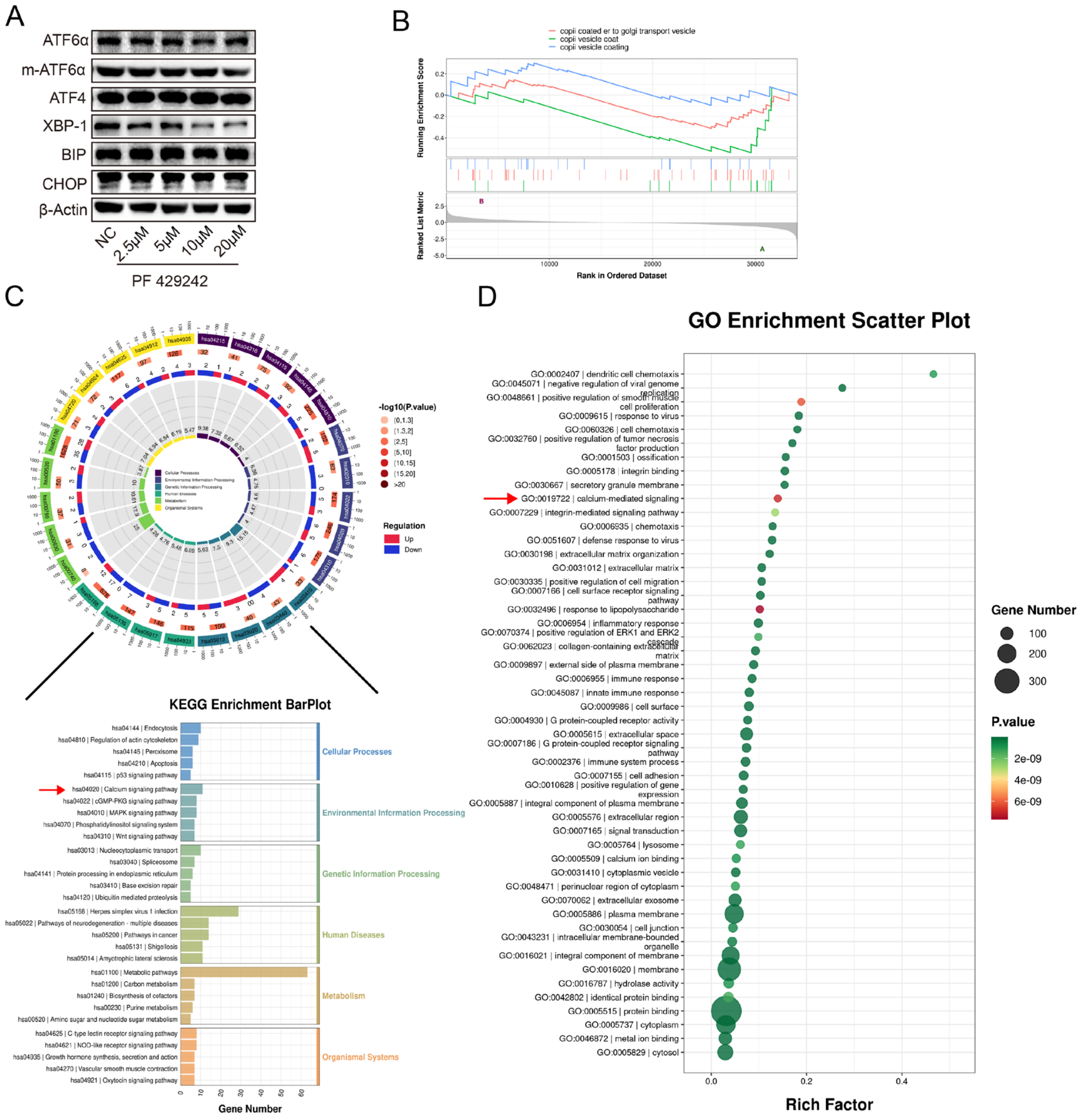

Supplemental Figure 4

(A) Immunoblots showing the gene expression of ATF6α, ATF4, BIP, XBP-1, CHOP in NP cells treated with different concentration of PF 429242. **(B)** Enriched pathways related to vesicle transport identified through Gene Set Enrichment Analysis (GSEA). **(C)** KEGG pathway enrichment plot, with red arrows indicating pathways related to calcium signaling. **(D)** GO enrichment scatter plot, with red arrows indicating pathways related to calcium signaling.

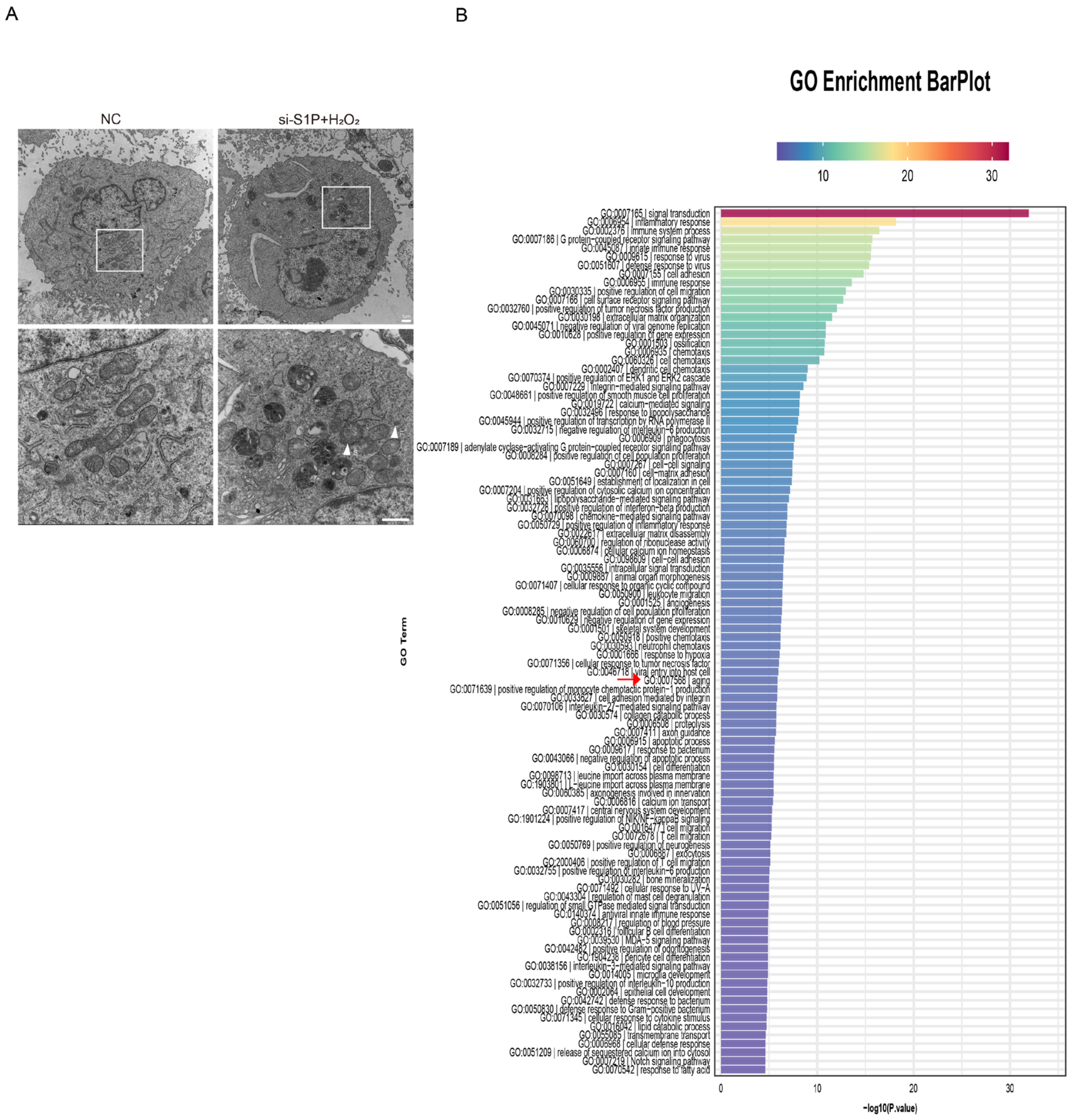

Supplemental Figure 5

(A) TEM images showing mitochondrial morphology. S: swollen mitochondria. F: fragmented mitochondria. Scar bars: 1 μm. **(B)** GO enrichment bar plot with red arrows indicating aging pathway.
