# Supplementary figures and images for: S1P regulates intervertebral disc aging by mediating endoplasmic reticulum–mitochondrial calcium ion homeostasis
Source: JCI Insight. 2024 Nov 8;9(21):e177789. doi: 10.1172/jci.insight.177789 (PMC11601718; doi:10.1172/jci.insight.177789)

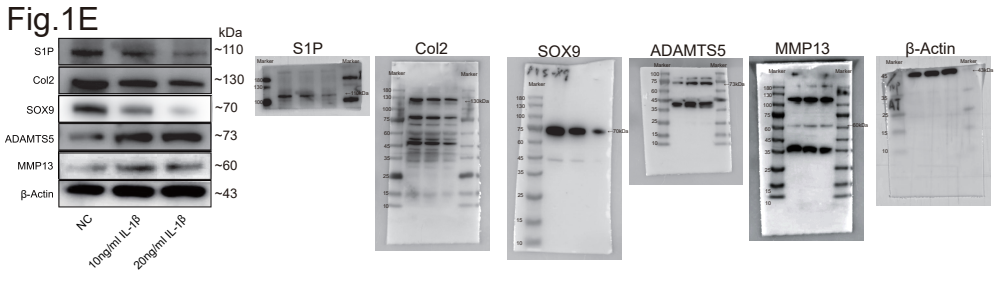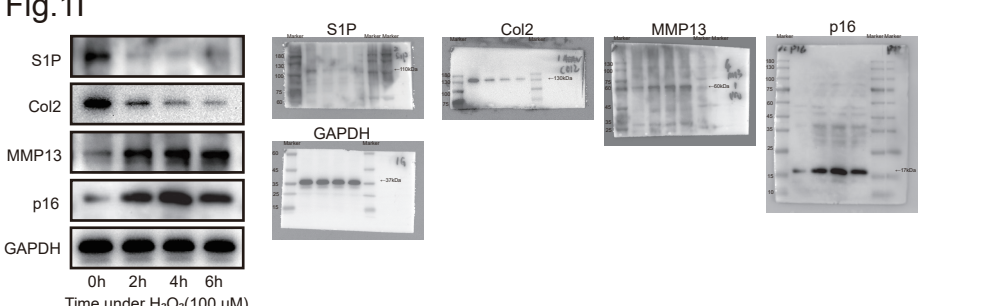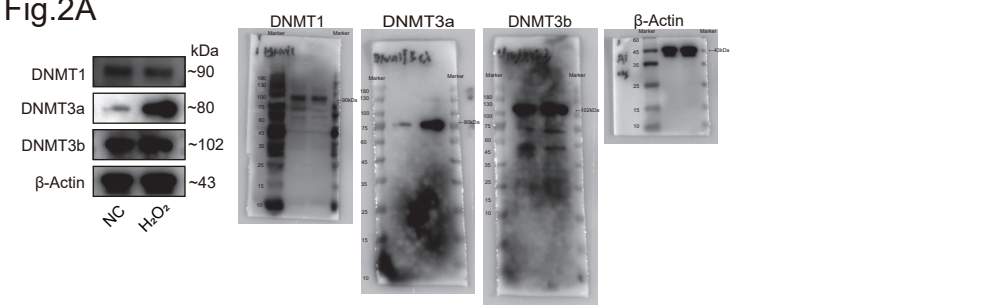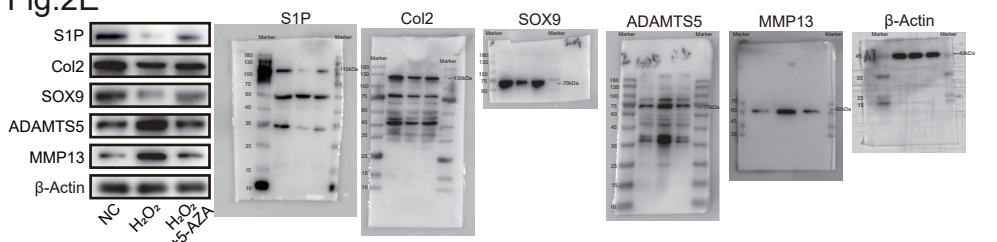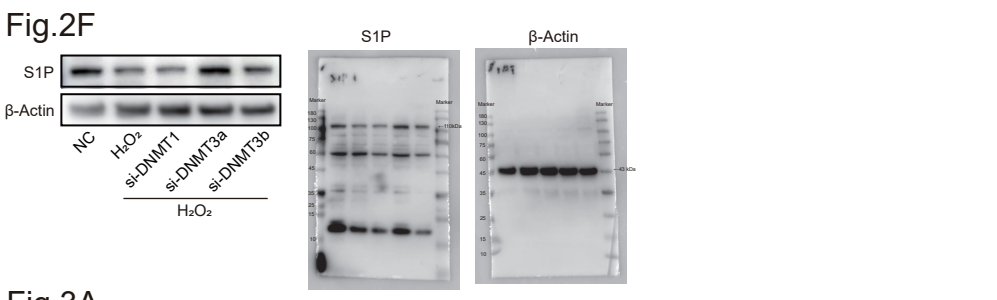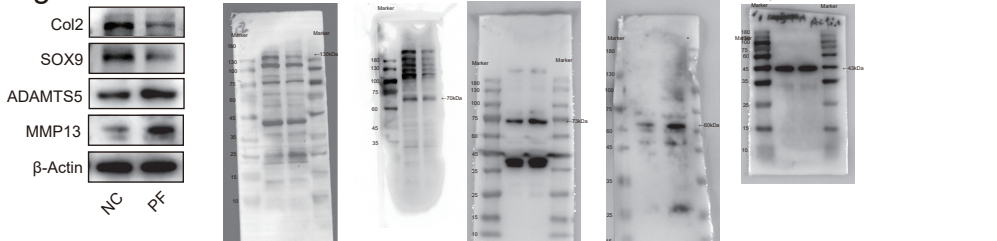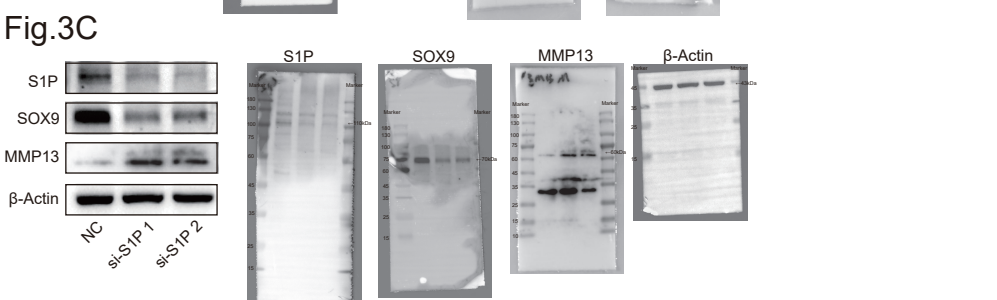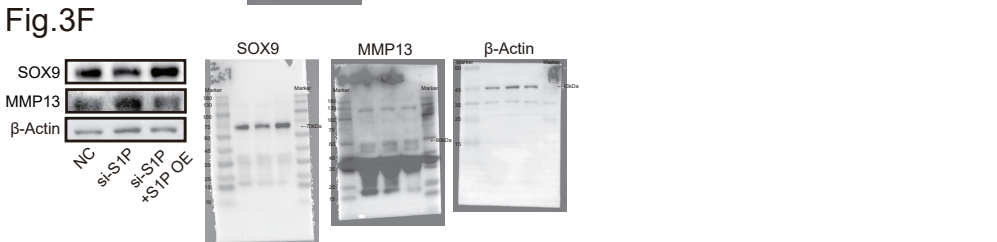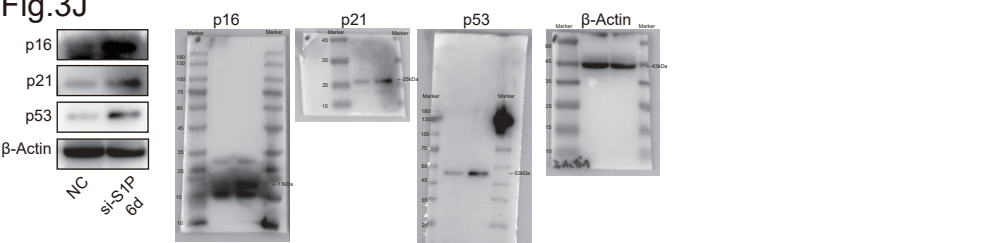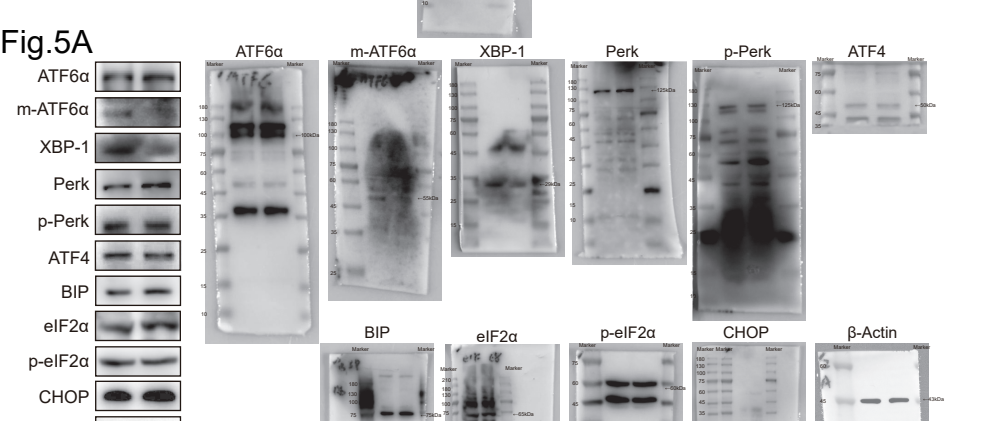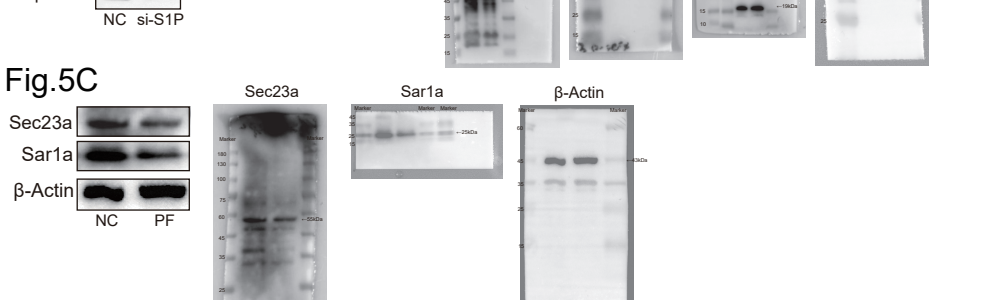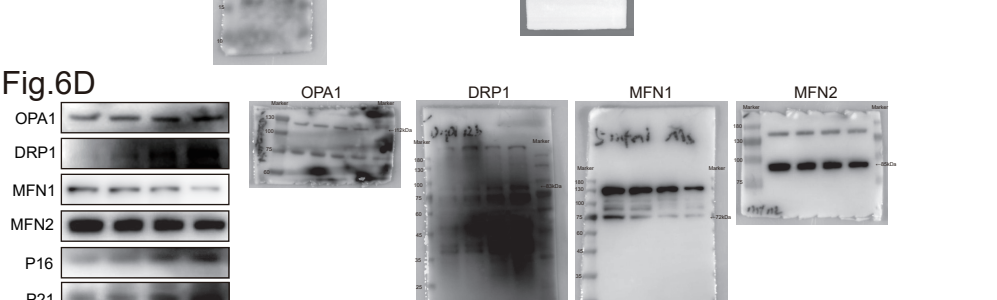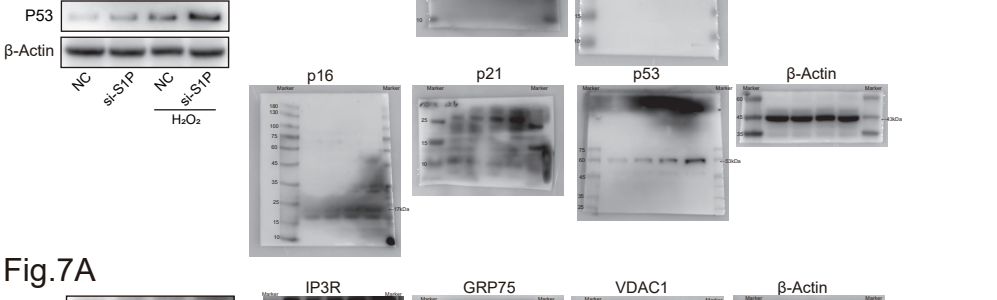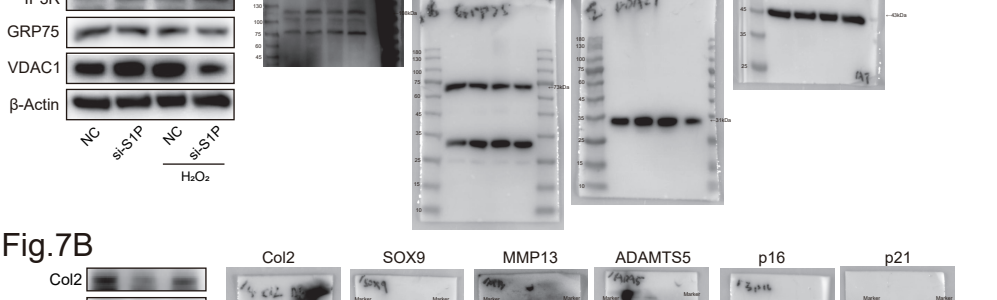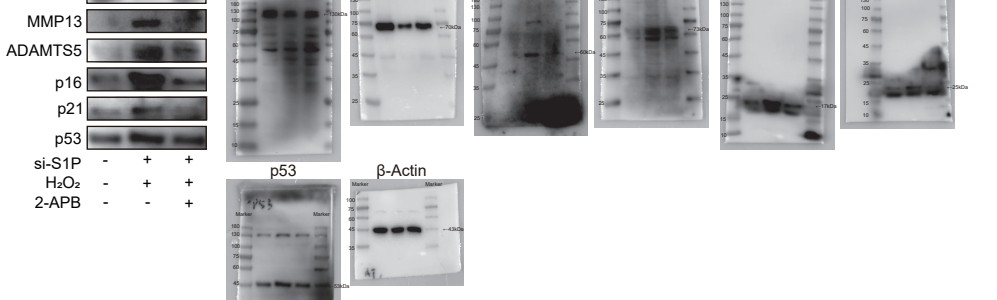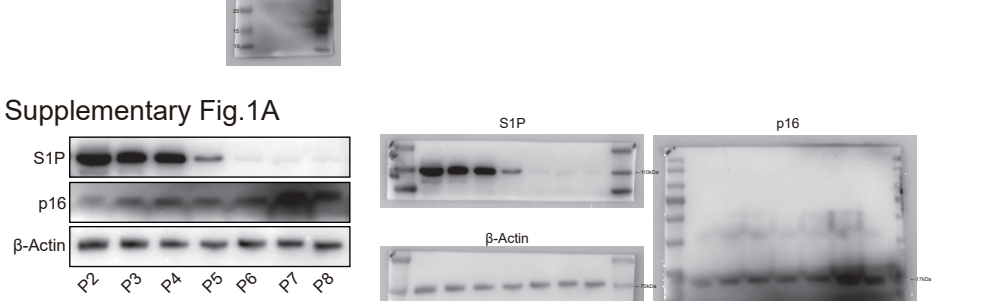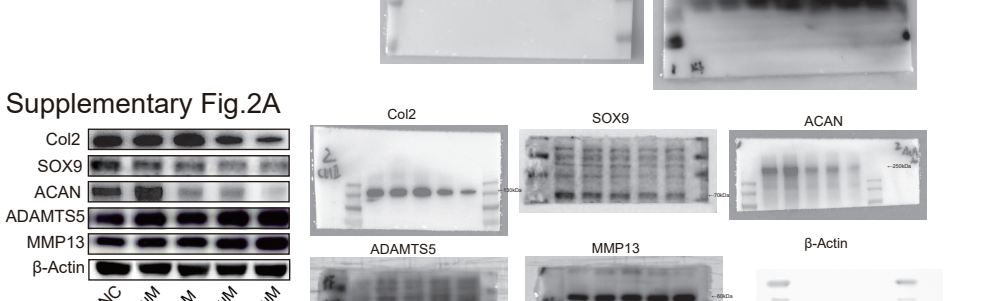

Supplement: Unedited blot and gel images [file jciinsight-9-177789-s089.pdf]
